# New bioactive metabolites from the elicited marine sponge-derived bacterium *Actinokineospora spheciospongiae* sp. nov.

**DOI:** 10.1186/s13568-018-0730-0

**Published:** 2019-01-24

**Authors:** Ahmed Tawfike, Eman Zekry Attia, Samar Yehia Desoukey, Dina Hajjar, Arwa A. Makki, Peter J. Schupp, RuAngelie Edrada-Ebel, Usama Ramadan Abdelmohsen

**Affiliations:** 1 Computational and Analytical Science Department, Rothamsted Research, AL5 2JQ Harpenden, UK; 2 Department of Pharmacognosy, Faculty of Pharmacy, Helwan University, Cairo, 11795 Egypt; 3 Department of Pharmacognosy, Faculty of Pharmacy, Minia University, Minia, 61519 Egypt; 4 Department of Biochemistry, Faculty of Science, Center for Science and Medical Research, University of Jeddah, 80203 Jeddah, Saudi Arabia; 5 Carl-von-Ossietzky University Oldenburg, Institute for Chemistry and Biology of the Marine Environment, Postfach 2503, 26111 Oldenburg, Germany

**Keywords:** Sponges, Actinomycetes, *Actinokineospora*, Fridamycin, Elicitation, Antitrypanosomal

## Abstract

Several approaches have been dedicated to activate the cryptic gene clusters in the genomes of actinomycetes for the targeted discovery of new fascinating biomedical lead structures. In the current study, *N*-acetylglucosamine was used to maximize the chemical diversity of sponge-derived actinomycete *Actinokineospora spheciospongiae* sp. nov. HR–ESI–MS was employed for dereplication study and orthogonal partial least square-discriminant analysis was applied to evaluate the HR–ESI–MS data of the different fractions. As a result, two new fridamycins H (**1**) and I (**2**), along with three known compounds actinosporin C (**3**), D (**4**), and G (**5**) were isolated from the solid culture of sponge-associated actinomycete *Actinokineospora spheciospongiae* sp. nov., elicited with *N*-acetylglucosamine. Characterization of the isolated compounds was pursued using mass spectrometry and NMR spectral data. Fridamycin H (**1**) exhibited significant growth inhibitory activity towards *Trypanosoma brucei* strain TC221. These results highlight the potential of elicitation in sponge-associated actinomycetes as an effective strategy for the discovery of new anti-infective natural products.

**Electronic supplementary material:**

The online version of this article (10.1186/s13568-018-0730-0) contains supplementary material, which is available to authorized users.

## Introduction

Marine sponge-derived actinomycetes have been recognized as potential producers of fascinating chemical scaffolds such as polyketides, alkaloids, peptides, sterols, terpenes and fatty acids (Abdelmohsen et al. 2014a, c; Genilloud 2017; Ibrahim et al. 2018; Van der Meij et al. 2017) with diverse pharmacological activities such as antichlamydial, antioxidant, antibacterial, antitrypanosomal, cytotoxic, and antibiofilm (Abdelmohsen et al. 2017; Balasubramanian et al. 2017; Cheng et al. 2015, 2017). The strain *Actinokineospora spheciospongiae* was first isolated by Abdelmohsen et al. in 2010 from the Red Sea sponge *Spheciospongia vagabunda* that was collected from Ras Mohamed, Egypt (Abdelmohsen et al. 2010). The strain was then described by Kampfer et al. in 2015 and the draft genome sequence was analysed (Harjes et al. 2014; Kampfer et al. 2015). The chemical and biological investigation of this strain emphasized the diversity of its natural components and resulted in identification of several new natural products (Abdelmohsen et al. 2014b; Dashti et al. 2017). Actinomycetes have been found to have the major fraction of their secondary metabolites to be transcriptionally inactive or “silent” under normal laboratory conditions (Abdelmohsen et al. 2015; Dinesh et al. 2017; Horn et al. 2015; Letzel et al. 2017; Seyedsayamdost 2014). In a study by Letzel et al. (2017), analysis of genome sequences of 119 marine actinomycetes reveals extraordinary biosynthetic diversity. Several experimental approaches have been applied to activate and identify the products of those cryptic genes using biological, chemical and genetic elicitations (Abdelmohsen et al. 2015; Dashti et al. 2017; Pettit 2011). In our previous work, *Actinokineospora spheciospongiae* broth culture was elicited with *N*-acetyl glucosamine (GlcNAc) and the metabolic profile was investigated using ^1^H NMR fingerprint methodology which resulted in identification of four new actinosporins (E–H) (Dashti et al. 2017). In the present study, the solid culture of *Actinokineospora spheciospongiae* sp. nov. was elicited with GlcNAc that led to the identification of two new fridamycins (**1** and **2**), along with three known compounds actinosporin C (**3**), D (**4**), and G (**5**). Moreover, the antitrypanosomal activity of all isolated pure compounds was also investigated.

## Materials and methods

### Microbial fermentation, extract preparation and compounds isolation

*Actinokineospora spheciospongiae* sp. nov. (DSM 45935T, GeneBank accession no. GU318361) was isolated from the Red Sea sponge *Spheciospongia vagabunda* that was collected by SCUBA diving from offshore Ras Mohamed, Egypt (GPS: 27°47.655 N; 34°12.904 W) in August 2006. Two hundred ISP2 agar plates (square 120 × 120 mm), each inoculated with 100 µL of a 5 day old liquid culture of *Actinokineospora spheciospongiae* and incubated at 30 °C for 6 h. The agar plates were then elicited with GlcNAc (50 µL/plate of a 50 μM solution, Sigma-Aldrich, Darmstadt, Germany). The plates were then incubated at 30 °C for 7 days. The agar medium with bacterial biomass was cut into small pieces and transferred to 1 L Erlenmeyer flasks. One liter of EtOAc/flask was added to submerge the agar pieces and macerate the medium culture under shaking at 150 rpm for overnight. The macerated medium was subsequently filtered using filter paper (A. Hartenstein, Wurzburg, Germany). The filtrates were combined and evaporated in a rotary evaporator under vacuum (Büchi, Essen, Germany) to yield 850 mg of dried crude EtOAc extract, which was fractionated on a Sephadex LH20 (50 g, 32–64 µm, 100 × 10 mm, Merck, Darmstadt, Germany) column eluting with H_2_O/MeOH (90:10%) to 100% methanol, to yield 12 fractions (50 mL each). The highly active fractions (3 and 5) were further purified by semi-preparative HPLC (Agilent, Waldbronn, Germany) using H_2_O/acetonitrile (95:5%) initially for 5 min, followed by a linear gradient to 100% acetonitrile within 40 min and maintained isocratically for further 5 min. Separation was achieved on a preparative C18 column (5 µm, 10 × 250 mm, Waters XBridge, Eschborn, Germany), with a flow rate of 3.0 mL/min yielding compounds **1** (1.6 mg) and **2** (1.8 mg) from fraction 3 and compounds **3**–**5** (2.3, 2.5 and 3 mg, respectively) from fraction 5.

### LC–MS analysis

One milligram of dried microbial extracts and/or fractions was dissolved in HPLC-grade MeOH to a final concentration of 1 mg/mL and subjected to high resolution LC–ESI–MS using Thermo Scientific Exact mass analysers (Thermo Scientific, Karlsruhe, Germany) coupled to a Dionex UltiMate 3000 HPLC system. The samples were eluted through a C-18 column (ACE, Mainz, Germany) with a length of 75 mm, internal diameter of 3.0 mm and particle size 5 μm. The mobile phase consisted of 0.1% formic acid in HPLC-grade water (solvent A) and acetonitrile (solvent B). The flow rate was set at 300 µL/min. Gradient elution was initiated with 10% B for 5 min, which was increased to 100% B over 30 min, which was maintained for another 5 min before decreasing to 10% B in the next min. The column was then equilibrated with 10% B for 4 min until the end of the run. MS/MS was measured using LTQ Orbitrap Thermo scientific mass analysers (Thermo Scientific, Karlsruhe, Germany).

### Biological activity of the isolated compounds

#### Antitrypanosomal activity

Antitrypanosomal action had been investigated following the method reported by Abdelmohsen et al. (2014b). In brief, Complete Baltz Medium was used for cultivation of 104 trypanosomes per mL of the *Trypanosoma brucei* strain TC 221 (Missionsärztliche Klinik, University of Wurzburg). 96-well plate chambers were used for testing Trypanosomes versus various concentrations of examined compounds at 0.25–40 μM in DMSO (1%) to a final volume of 200 μL. For controls, DMSO (1%) plus parasites without any examined compound were used concurrently in each plate. After that, the plates were incubated in an atmosphere of CO_2_ 5% at 37 °C for 24 h. Then, 20 μL of Alamar Blue was added in each plate and the activity was measured by light absorption at ʎ 550 nm with a reference wavelength of 650 nm using an MR 700 Microplate Reader (Dynatech Engineering Ltd., Willenhall, UK) after 48 and 72 h. The quantification of the IC_50_ values of the examined compounds were processed by linear interpolation in triplicate.

#### Cytotoxicity activity

Macrophages (J774.1) (Missionsärztliche Klinik, University of Wurzburg) were cultivated in Complete Baltz Medium free from phenol red. Different concentrations of tested compounds (0.25–200 μM) at a cell density of 1 × 10^5^ cells/mL at 37 °C, 5% CO_2_, and 95% humidity for 24 h, were used for investigation of their cytotoxic properties against Macrophages (J774.1). After adding of Alamar Blue (20 μL), the plates were incubated and the then, determination of the optical densities at ʎ 540 nm and a reference wavelength of 630 nm were done after 48 and 72 h, in the same way as described for the anti-trypansomal activity (Abdelmohsen et al. 2014b).

#### Compounds characterization

Fridamycin H (**1**): Orange powder; [α]_D_^25^ + 12° (*c* 1.5, MeOH); UV (MeOH) λ max (log ε) 231 (4.45), 253 (4.51), 293 (4.37) nm; IR (KBr)_νmax_ 3420, 2962, 1710, 1436, 1340, 1275, 1065 cm^−1^; ^1^H NMR (DMSO-d_6_, 600 MHz) and ^13^C NMR (DMSO-d_6_, 150 MHz) data, see Table 2; HR-ESIMS (+) *m/z* 503.1524 [M+H]^+^ (calcd. for C_25_H_26_O_11_, 503.1553).

Fridamycin I (**2**): Yellow powder; [α]_D_^25^ + 25° (*c* 1.5, MeOH); UV (MeOH) λ max (log ε) 232 (4.54), 253 (4.53), 294 (4.17) nm; IR (KBr)_νmax_ 3419, 3030, 1700, 1650, 1432, 1345, 1275, 1065 cm^−1^; ^1^H NMR (DMSO-d_6_, 600 MHz) and ^13^C NMR (DMSO-d_6_, 150 MHz) data, see Table 3; HR-ESIMS (+) *m/z* 614.2036 [M+Na]^+^ (calcd. for C_32_H_33_NO_10_Na, 614.2021).

## Results

### Bioactivity-guided assay and metabolomic analysis

**Fig. 1:**
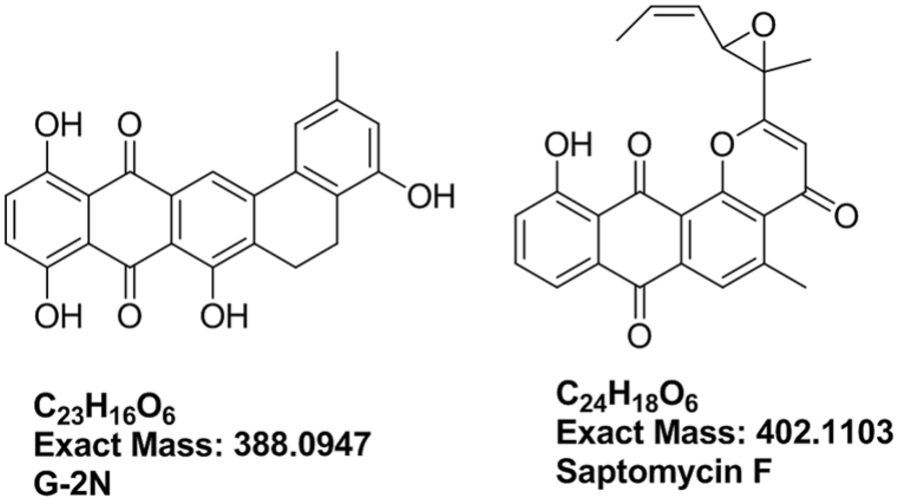
Dereplicated structures from the active fractions: G-2N and saptomycin F

All fractions were subjected to HPLC-HR-ESIMS analysis on both negative and positive ionization switch modes to cover the maximum number of metabolites. The MS dataset was processed and data were extracted using MZmine 2.20 based on the established parameters (Tawfike et al. 2017). The dereplication study of the active fractions against DNP database revealed the presence of known metabolites, but mostly unknown hits dominated as shown in Table 1. Metabolites at *m/z* (retention time in minutes) 387.0865 (6.65) [M−H]^−^ and 401.1025 (10.44) [M−H]^−^, were dereplicated as G-2N (C_23_H_16_O_6_), an antibiotic formerly reported from actinomycete *Frankia* sp.(Nancy and Mary 1984), and saptomycin F (C_24_H_18_O_6_), an antibiotic previously isolated from *Streptomyces* sp.(Abe et al. 1993), respectively. The aforementioned dereplicated metabolites, depicted in Fig. 1, possessed the naphthacene-dione moiety. Additionally, the mass ion peak at *m/z* 227.175 [M+H]^+^ for the predicted molecular formula C_12_H_22_N_2_O_2_ was dereplicated as isoleucyl isoleucine anhydride. The latter is a dipeptide previously reported from *Ustilago cynodontis* and *Beauveria bassiana* (Geraci et al. 2000).

**Table 1:** The dereplication results of the active fractions against DNP database

Polarity	m/z	Rt	Formula	Name	Source
[M−H]^−^	387.0865	6.65	C_23_H_16_O_6_	G-2N	*Frankia* sp. ANP 190107
[M+H]^+^	188.1279	7.69	C_9_H_17_NO_3_	Unknown	–
[M−H]^−^	343.0952	8.67	C_18_H_12_N_6_O_2_	Unknown	–
[M+H]^+^	227.1753	9.00	C_12_H_22_N_2_O_2_	Isoleucyl isoleucine anhydride	*Ustilago cynodontis*, *Beauveria bassiana*
[M−H]^−^	369.0751	9.84	C_19_H_10_N_6_O_3_	Unknown	–
[M−H]^−^	1003.325	9.86	–	Complex of 501.1586 and 501.1586 m/z	
[M−H]^−^	501.1586	9.86	C_31_H_18_N_8_	Unknown	–
[M+H]^+^	357.097	9.88	C_19_H_16_O_7_	Fridamycin E	*Streptomyces parvulus* Tu 1989
[M−H]^−^	401.1025	10.44	C_24_H_18_O_6_	Saptomycin-F	*Streptomyces* strain HP530
[M+H]^+^	357.0968	15.66	C_16_H_8_N_10_O	Unknown	–
[M−H]^−^	355.0954	15.67	C_19_H_12_N_6_O_2_	Unknown	–

The dereplication results highlighted the previously reported active compounds that would guide the structure elucidation of the isolated unknown metabolites. Multivariate data analysis (MVDA) was performed for the MS dataset of active versus inactive fractions to pinpoint the putatively active metabolites in the antitrypanosomally-active fractions prior to any further purification attempts.

**Fig. 2:**
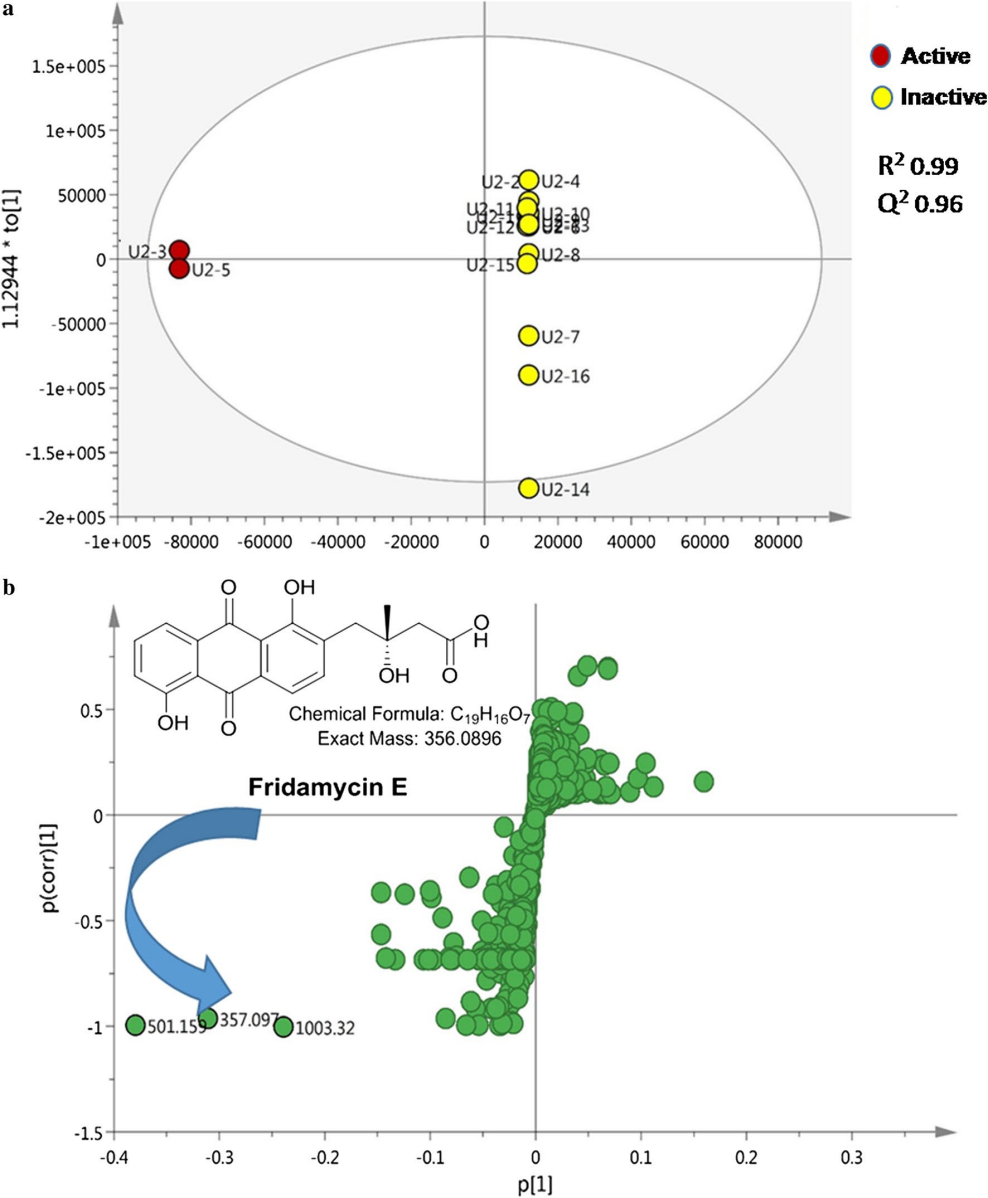
**a** OPLS-DA score plot of active (red circle) versus inactive fractions (yellow circle). **b** S-loading plot showing the putatively active metabolites

To achieve the best discrimination between the active and inactive fractions, a supervised orthogonal partial least square discriminant analysis (OPLS-DA) was carried out. The OPLS-DA scores plot (Fig. 2a) showed a significant discrimination between active and inactive fractions revealing a regression fit of 99% (R^2^ = 0.99) and high predictive ability of Q^2^ = 0.96. The current OPLS-DA model produced the highest predictive power (Q^2^) when assessed by cross validation. Moreover, the statistical significance of estimated predictive power of the respective model was tested by permutation test which exhibited Q^2^Y intercept at − 0.81 that indicated a valid model. A particularly useful tool that compares the variable magnitude against its reliability is the S-loadings plot obtained from the OPLS-DA model and represented in Fig. 2b, where axes plotted from the predictive component are the covariance p[1] against the correlation p (cor) [1]. The S-loadings plot highlighted the metabolites distinct for the active fractions that highly correlated to their bioactivity.

The metabolite at *m/z* 501.159 (Rt = 9.86 min) [M−H]^−^, equivalent to C_25_H_26_O_11_ did not match any compounds reported in the DNP database while the *m/z* ion at 357.097 (Rt = 9.88 min) [M+H]^+^ for the predicted molecular formula C_19_H_16_O_7_ was dereplicated for fridamycin E, the simplest member of the angucycline family of antibiotics that was previously isolated from a mutant of *Streptomyces parvulus* (strain Tu 1989) (Krohn and Baltus 1988; Kunzel et al. 1999). The metabolomics and dereplication results (Table 1, Fig. 1) revealed previously undescribed putatively active metabolites that motivated us to further purify the fractions and identify the active compounds via ^1^D, ^2^D-NMR and HR–ESIMS.

### Structure elucidation of the new isolated compounds

**Fig. 3:**
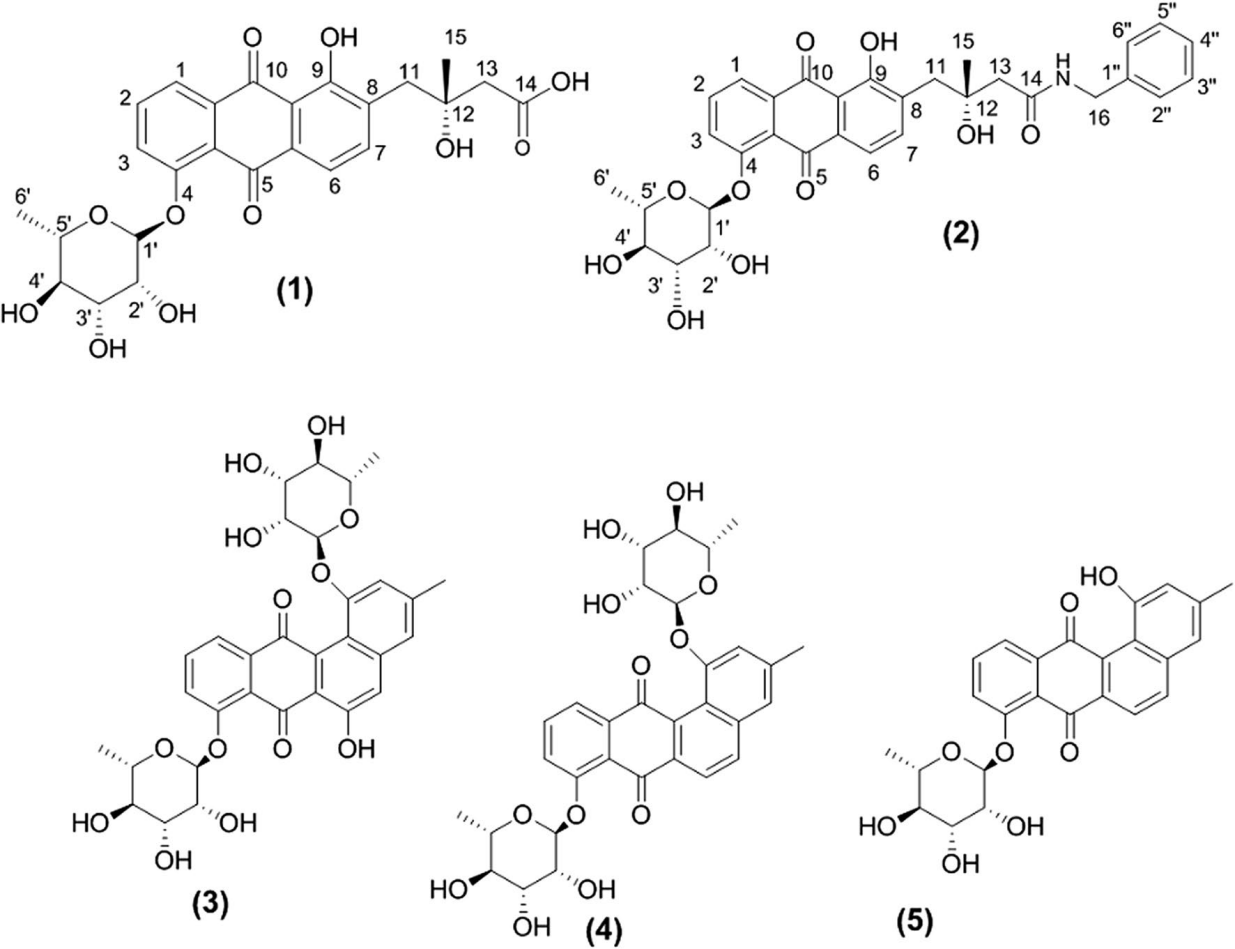
Structures of the isolated compounds: fridamycin H (**1**) and I (**2**), actinosprin C (**3**), D (**4**) and G (**5**)

Separation of fraction 3 delivered two hydroxyquinones with similar UV spectra (231, 253, 293 nm) indicating similar chromophores. A molecular formula of C_25_H_26_O_11_ was established for **1** (Fig. 3) by positive HR-ESIMS analysis (found at *m/z* 503.1524 for [M+H]^+^, calcd. 503.1553) requiring thirteen degrees of unsaturation (Additional file 1: Figure S1). The 600 MHz instrument for ^1^H, ^13^C, COSY, HSQC and HMBC (optimized for *J* = 8.3 Hz and *J* = 4.0 Hz) in DMSO-d_6_ was used for processing the NMR spectral analyses. Initial investigation of NMR spectral data of **1** (Table 2) exhibited characteristic signals for a C-4 substituted (*R*)-fridamycin E moiety (Krohn and Baltus 1988; Myronovskyi et al. 2016; Vicente et al. 2015).

**Table 2:** ^1^H and ^13^C NMR data (DMSO-d_6_, 600 and 150 MHz) of **1**

Position	δ_H_, mult (*J* in Hz)	δ_C_, type	COSY	HMBC
1	7.95, d (7.8)	120.6, CH	H-2	C-3, 10
2	7.86, dd (7.8, 8.4)	135.4, CH	H-1, 3	C-4, 10a
3	7.70, d (8.4)	123.6, CH	H-2	C-1, 4a
4	–	156.8, C	–	–
4a	–	122.3, C	–	–
5	–	181.2, C	–	–
5a	–	133.4, C	–	–
6	7.61, d (7.8)	118.2, CH	H-7	C-8, 9a, 5
7	7.78, d (7.8)	141.0, CH	H-6	C-5a, 9
8	–	133.4, C	–	–
9	–	160.3, C	–	–
9a	–	115.1, C	–	–
10	–	188.9, C	–	–
10a	–	135.2, C	–	–
11a 11b	2.88, d (13.2) 3.03, d (13.2)	40.1, CH_2_	–	C-7, 9
12	–	71.5, C	–	–
13	2.40, s	46.7, CH_2_	–	C-11
14	–	172.8, C	–	–
15	1.19, s	26.8, CH_3_	–	C-11, 13
1′	5.64, d (1.8)	99.3, CH	H-2′	C-4′, 5′
2′	3.99, m	70.5, CH	H-1′	C-1′
3′	3.99, m	70.5, CH	H-4′	–
4′	3.33, m	72.0, CH	H-3′	–
5′	3.52, m	70.4, CH	H-4′, 6′	–
6′	1.09, d (6)	18.4, CH_3_	H-5′	C-5′, 4′

The aromatic region in ^1^H NMR spectrum (Additional file 1: Figure S2) displayed the presence of two different aromatic spin systems of *ortho*-coupled protons; one corresponds to a tri-substituted benzene ring consisting of two one-proton doublets at δ_H_ 7.95 (*J* = 7.8 Hz) and 7.70 (*J* = 8.4 Hz) assignable to H-1 and H-3, respectively, in addition to a one proton doublet of doublet at δ_H_ 7.86 (*J* = 7.8 and 8.4 Hz), of H-2. Another spin system consists of two one-proton doublets at δ_H_ 7.78 (*J* = 7.8 Hz) and 7.61 (*J* = 7.8 Hz) assignable to protons H-6 and H-7, respectively, of the tetra-substituted aromatic system. Moreover, the ^1^H NMR spectrum showed two one-proton doublets at δ_H_ 2.88 (*J *= 13.2 Hz) and 3.03 (*J *= 13.2 Hz), attributed to the two geminally coupled protons, characteristic for H-11a and H-11b, respectively. Whereas, the two-protons singlet at δ_H_ 2.40 was assignable for H-13, belonging to the protons of a methylene group directly linked to the carboxyl group. One singlet at δ_H_ 1.19 together with its corresponding carbon signal at δ_C_ 26.8 was for the methyl group (CH_3_-15) which was considerably downfield shifted due to its direct attachment to oxygenated carbon at δ_C_ 71.5 (C-12) (Vicente et al. 2015). Moreover, the presence of an anomeric proton signal at δ_H_ 5.64 (*J* = 1.8 Hz; δC 99.3), one methyl doublet at δ_H_ 1.09 (*J *= 6 Hz), as well as four one-proton multiplets at δ_H_ (3.33–3.99) indicated the presence of the rhamnose moiety in the structure (Grkovic et al. 2014). The coupling constant of the anomeric proton allowed the identification of an α anomer and the absolute configuration of sugar was determined to be L based on the literature (Dashti et al. 2017).

**Fig. 4:**
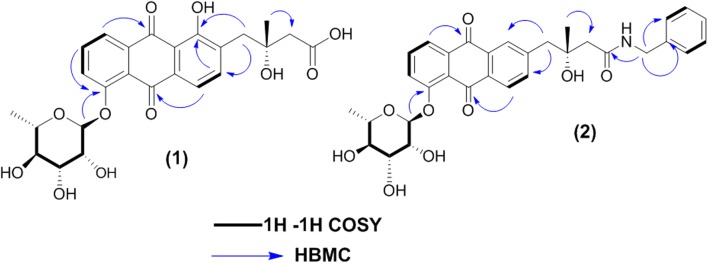
Key COSY and HMBC correlations of fridamycin H **(1)** and I (**2**)

The ^13^C NMR and HSQC spectra (Additional file 1: Figures S3, S5) displayed fourteen resonances comprising five methine sp^2^, five methine sp^3^, two methylene sp^3^, two methyl carbons consistent with an angucycline core and one glycosidic residue. Three carbonyl carbons were displayed, the upfield shifted carbon at δ_C_ 172.8 was attributed to free carboxyl group (C-14), while the others resonated at δ_C_ 181.2 and 188.9 were assigned to quinone carbonyl carbons C-5 and C-10, respectively. Additionally two methylene carbons C-11 and C-13 were observed; the latter was deshielded to 46.7 ppm in agreement with direct attachment to carboxylic group, while the former one was detected at 40.1 ppm due to its direct attachment to the aromatic ring that could be confirmed by HMBC correlations from H-11a and H-11b (δ_H_ 2.88 and 3.03) to C-7 (δ_C_ 141.0) and C-9 (δ_C_ 160.3). Two oxygenated aromatic carbons were observed at δ_C_ 156.8 and 160.3, assignable to C-4 and C-9, respectively. The former (C-4) displayed HMBC correlation to the anomeric proton of l-rhamnose (δ_H_ 5.64, H-1′), confirming the attachment of l-rhamnose to C-4 of the anthraquinone motif (Fig. 4). Due to a small amount of **1** obtained in this study, it was not possible to determine the absolute configuration of C-12. However, as **1** is a 4-*O*-glycosylated fridamycin E, we speculated that the absolute configuration of C-12 of **1** should be the same as that of naturally occurring fridamycin E (Krohn and Baltus 1988) i.e. 12R. However, it is necessary to obtain more amount of **1** to determine conclusively the absolute configuration of C-12. As a common biosynthesis feature of *Streptomyces* sp., all the naturally identified fridamycin E analogues retain the (R) configuration (α-OH and β-CH_3_) at C-12. Whereas the (S) isomers, (α-CH_3_ and β-OH) at C-12, have been only obtained chemosynthetically as the less bioactive isomers of fridamycin E analogues (Bruntner et al. 2005; Chen et al. 2011; Rohr and Thiericke 1992; Vicente et al. 2015).

COSY, HSQC and HMBC experiments (Additional file 1: Figures S4–S6) were used to support the aforementioned assignments and the structure of **1**, which was a new compound that we named fridamycin H.

HR-ESIMS of **2** (Fig. 3) established the molecular formula as C_32_H_33_NO_10_Na (found at *m/z* 614.2036, calcd. 614.2021) [M+Na]^+^) requiring seventeen degrees of unsaturation Additional file 1: Figure S7). First investigation of the NMR spectroscopic data of **2** (Table 3) (Additional file 1: Figures S8–S12) showed many signals similar to chemical shifts of those reported for **1** (fridamycin H) except for the substitution of the carboxyl group of **1** with *N*-benzyl amide moiety that was represented by multiplet signals at δ_H_ 7.25, 7.30 and 7.24 assignable to (H-2″, 6″), (H-3″, 5″) and H-4″, respectively, in accordance with mono-substituted benzene ring. Two-protons singlet at δ_H_ 3.54 along with its corresponding carbon signal at δ_C_ 40.9 were for the methylene group (CH_2_-16) directly attached to the benzene ring and one singlet at δ_H_ 8.17 corresponding to an amine proton (Myronovskyi et al. 2016). Moreover, HMBC correlations from H-16 (δ_H_ 3.54) to C-1″ and C-2″, 6″ at δ_C_ 135.45 and 129.7, respectively and to amide-type carbonyl carbon at (δ_C_ 173.2) were observed confirming direct attachment of the methylene group (CH_2_-16) to the benzene ring from one side and to the amide moiety from the other side (Myronovskyi et al. 2016) (Fig. 4).

**Table 3:** ^1^H and ^13^C NMR data (DMSO-d_6_, 600 and 150 MHz) of **2**

Position	δ_H_, mult (*J* in Hz)	δ_C_, type	COSY	HMBC
1	7.95, d (7.8)	120.6, CH	H-2	C-3, 4a, 10
2	7.85, dd (7.8, 8.4)	135.5, CH	H-1, 3	C-4, 10a
3	7.70, d (8.4)	123.8, CH	H-2	C-1, 4a
4	–	156.7, C	–	–
4a	–	122.1, C	–	–
5	–	180.8, C	–	–
5a	–	133.35, C	–	–
6	7.61, d (7.8)	118.3, CH	H-7	C-8, 9a, 5
7	7.79, d (7.8)	140.3, CH	H-6	C5a, 9, 11
8	–	133.2, C	–	–
9	–	160.3, C	–	–
9a	–	115.1, C	–	–
10	–	189.0, C	–	–
10a	–	135.3, C	–	–
11a 11b	2.88, d (13.2) 3.03, d (13.2)	39.9, CH	–	C-7, 9, 13, 15
12	–	71.4, C	–	–
13	2.39, s	46.6, CH_2_	–	C-11, 15
14	–	173.2, C	–	–
15	1.18, s	26.5, CH_3_	–	C-11, 13
16	3.54, s	40.9, CH_2_	–	C-14, 1″, 2″, 6″
1′	5.64, d (1.8)	99.0, CH	H-2′	C-4, 5″
2′	3.99, m	70.4, CH	H-1′	C-1″
3′	3.99, m	70.5, CH	H-4′	–
4′	3.33, m	72.0, CH	H-3′	–
5′	3.52 m	70.6, CH	H-4′, 6′	–
6′	1.08, d (6)	18.2, CH_3_	H-5′	C-5″, 4″
1″	–	135.5, C	–	–
2″, 6″	7.25, m	129.7, CH	H-3″, 5″	–
3″, 5″	7.30, m	128.7, CH	H-2″, 6″	–
4″	7.24 m	127.0, CH	–	–
14-NH	8.17 br s	–	–	–

COSY, HSQC and HMBC experiments (Additional file 1: Figures S10–S12) were used to support the aforementioned assignments and the structure of compound **2**, which was a new compound that we named fridamycin I.

Separation of fraction 5 delivered three actinosporins with similar UV spectra (250, 270, and 320 nm). By comparing their spectra (Additional file 1: Figures S13–S21) with previously published data (Dashti et al. 2017; Grkovic et al. 2014), the structures of these compounds were confirmed as actinosporin C (**3**), D (**4**) and G (**5**).

### Biological activity of the isolated compounds

All the isolated compounds were tested against *Trypanosoma brucei* strain TC221 (Abdelmohsen et al. 2014b; Huber and Koella 1993). Compound **1** showed significant antitrypanosomal activity after 48 and 72 h with IC_50_ values of 7.18 and 3.35 μM, respectively, with no cytotoxicity against J774.1 macrophages (IC_50_ of > 200 μM). While the rest displayed no significant antitypanosomal or cytotoxic activity at the concentration tested.

## Discussion

Bioinformatic analyses have revealed the presence of large fractions of silent gene clusters in the genome sequences of actinomycetes. Innovative strategies had been used to induce production of weakly expressed secondary metabolites which are not expressed under standard fermentation conditions and several approaches including biological, chemical, and molecular, have been applied to activate those silent natural products in the laboratory culture of actinomycetes (Abdelmohsen et al. 2015). Among those approaches, OSMAC approach (one strain many compounds), co-cultivation and applying of external chemical elicitors (Romano et al. 2018). In our previous studies the genome sequences (Harjes et al. 2014) as well as metabolomic analyses (Abdelmohsen et al. 2014b) of *Actinokineospora spheciospongiae* sp. nov. showed unexplored chemical potential. Actinosporins A and B had been isolated using broth culture of *Actinokineospora* sp. strain EG49 (Abdelmohsen et al. 2014b), while supplementing the same culture with calcium alginate beads allowed identification of actinosporins C and D have been identified (Grkovic et al. 2014). Moreover, induction of liquid culture of *Actinokineospora* sp. EG49 with GlcNAc has resulted in the isolation of and characterization of actinosporins E–H (Dashti et al. 2017). In the current approach, elicitation of the solid culture of *Actinokineospora spheciospongiae* sp. nov. via GlcNAc resulted in isolation and characterization of two new fridamycins H (**1**) and I (**2**), along with three known actinosporins C (**3**), D (**4**) and G (**5**).

On the other side, trypanosomiasis, commonly known as sleeping sickness, still has been considered one of the most serious challenges to human health, particularly in underdeveloped regions of Africa (Jacobs et al. 2011; Malvy and Chappuis 2011). It is commonly combined with degradation of hepatic cells, anemia and glomerulonephritis which are likely ascribed to considerable free radicals produced by the parasites which attack cellular membrane, leading to cellular damages of human vital organs. So far, effective antitrypanosomally drugs are still not accessible, regardless the extensive efforts to achieve treatments (Ibrahim et al. 2013; Steverding 2010). Thus, ongoing research for new classes of metabolites with antitrypanosomal activity is necessary. Consequently, two new fridamycins H (**1**) and I (**2**), along with three known actinosporins C (**3**), D (**4**) and G (**5**) were considered for the examination of the antitrypanosomal property versus *Trypanosoma brucei* TC221 in addition to cytototoxic properties towards Macrophages (J774.1). The results obtained displayed that fridamycin H showed significant antitrypanosomal activity after 48 and 72 h with IC_50_ values of 3.38 and 5.26 μM, respectively, with no cytotoxicity against J774.1 macrophages (IC_50_ > 200 μM). These results demonstrate the potential of elicitation via compound induction as a tool for discovery of new antitrypanosomal chemical scaffolds.

## Additional file

**Additional file 1.** Additional figures.
