# Supplementary material for: New bioactive metabolites from the elicited marine sponge-derived bacterium Actinokineospora spheciospongiae sp. nov
Source: AMB Express. 2019 Jan 24;9:12. doi: 10.1186/s13568-018-0730-0 (PMC6345950; doi:10.1186/s13568-018-0730-0)
Supplement: Supplementary file 1 — Additional file 1. Additional figures. [file 13568_2018_730_MOESM1_ESM.pdf]

Applied Microbiology and Biotechnology Express

New bioactive metabolites from the elicited marine sponge-derived bacterium *Actinokineospora spheciospongiae* sp. nov.

Ahmed Tawfike[1, 2], Eman Zekry Attia[3], Samar Yehia Desoukey[3], Dina Hajjar[4], Arwa A Makki[4], Peter J. Schupp[5*], RuAngelie Edrada-Ebel[1], and Usama Ramadan Abdelmohsen[3*]

1 Strathclyde Institute of Pharmacy and Biomedical Sciences, University of Strathclyde, The John Arbuthnott Building, 27 Taylor Street, G4 0NR Glasgow, UK; ahmed.tawfike@rothamsted.ac.uk (A.T);ruangelie.edrada-ebel@strath.ac.uk (R. E)

2 Department of Pharmacognosy, Faculty of Pharmacy, Helwan University, Cairo11753, Egypt

3 Department of Pharmacognosy, Faculty of Pharmacy, Minia University, Minia 61519, Egypt; eman_zekry@mu.edu.eg (E.Z.A); drsamaryehia@gmail.com (S.Y.D); usama.ramadan@mu.edu.eg (U.R.A)

4 Department of Biochemistry, faculty of Science, Center for Science and medical research, University of Jeddah, 80203 Jeddah, Saudi Arabia; Dhajjar@uj.edu.sa (D.H); amaki@uj.edu.sa (A.A.M)

5 Carl-von-Ossietzky University Oldenburg, Institute for Chemistry and Biology of the marine Environment, Postfach 2503, 26111 Oldenburg, Germany; peter.Schupp@uni-oldenburg.de

* Correspondence: usama.ramadan@ mu.edu.eg, Tel.: +2-86-2347759, Fax: +2-86-2369075 (URA); peter.schupp@uni-oldenburg.de, Tel.: ++49 (0) 4421- 944-100, Fax: +49 (0) 4421- 944-140 (PJS)

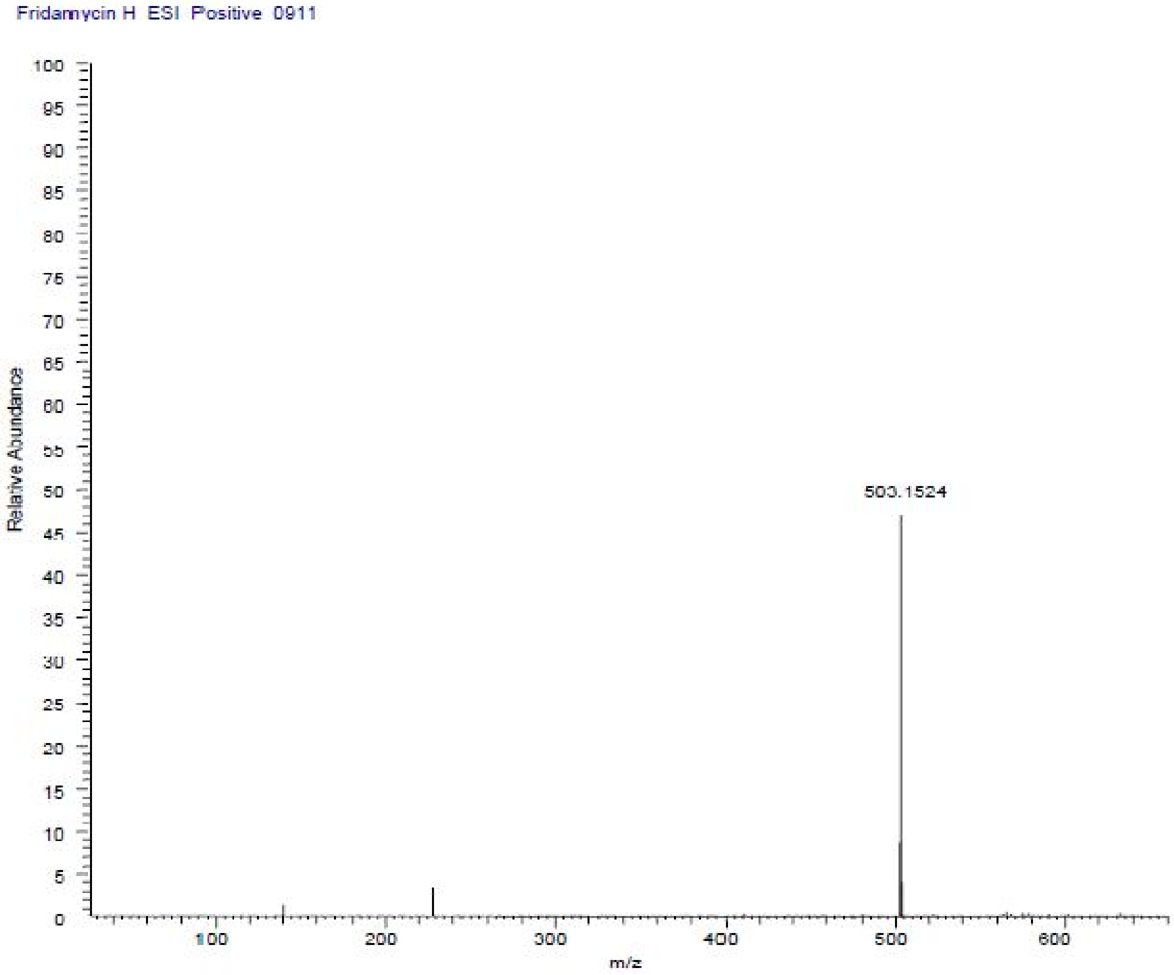

Figure S1. HR-ESIMS spectrum of compound 1 (Fridamycin H)

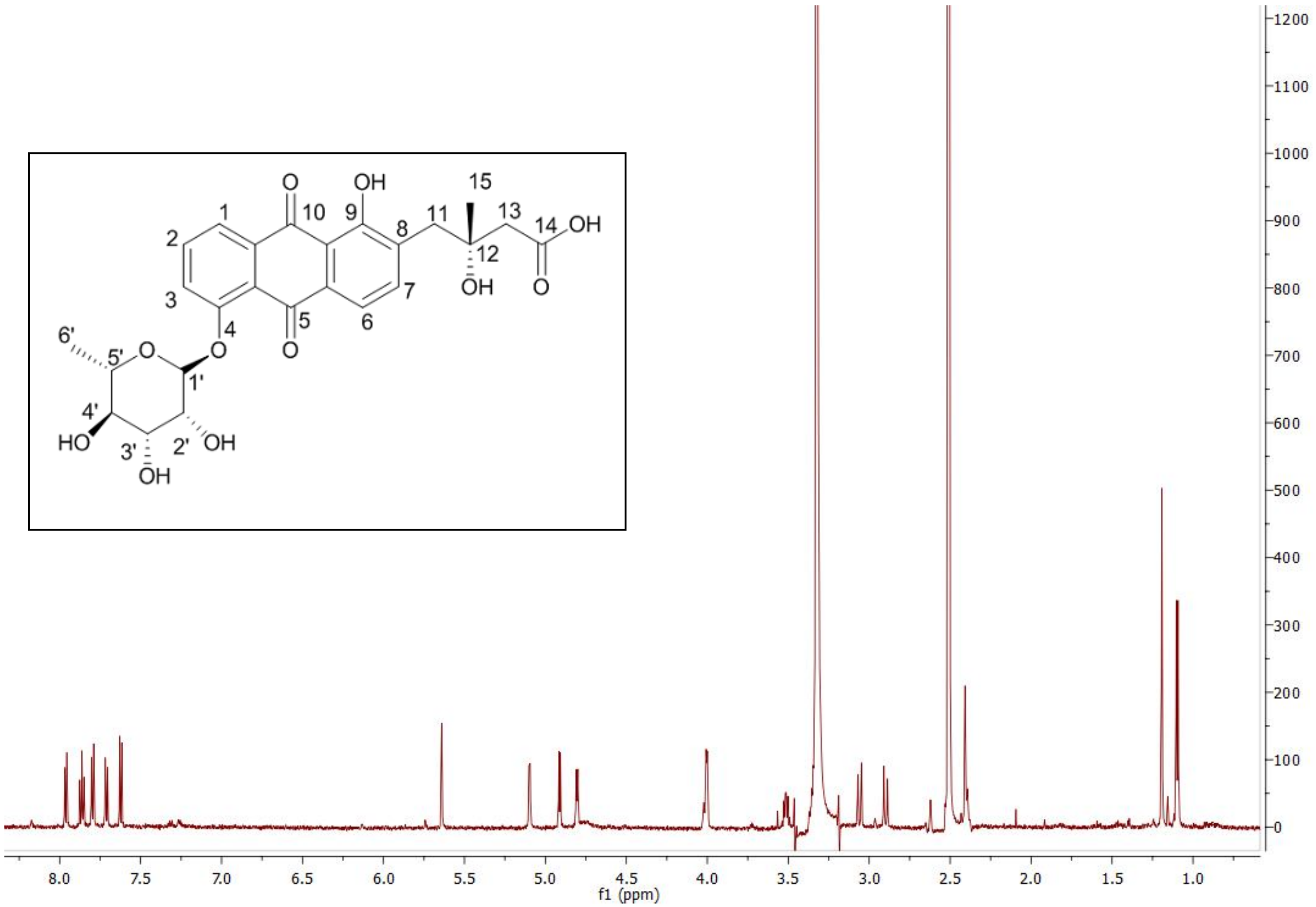

Figure S2. ^{1}H-NMR spectrum of compound 1 (Fridamycin H) in DMSO-*d6* (600 MHz)

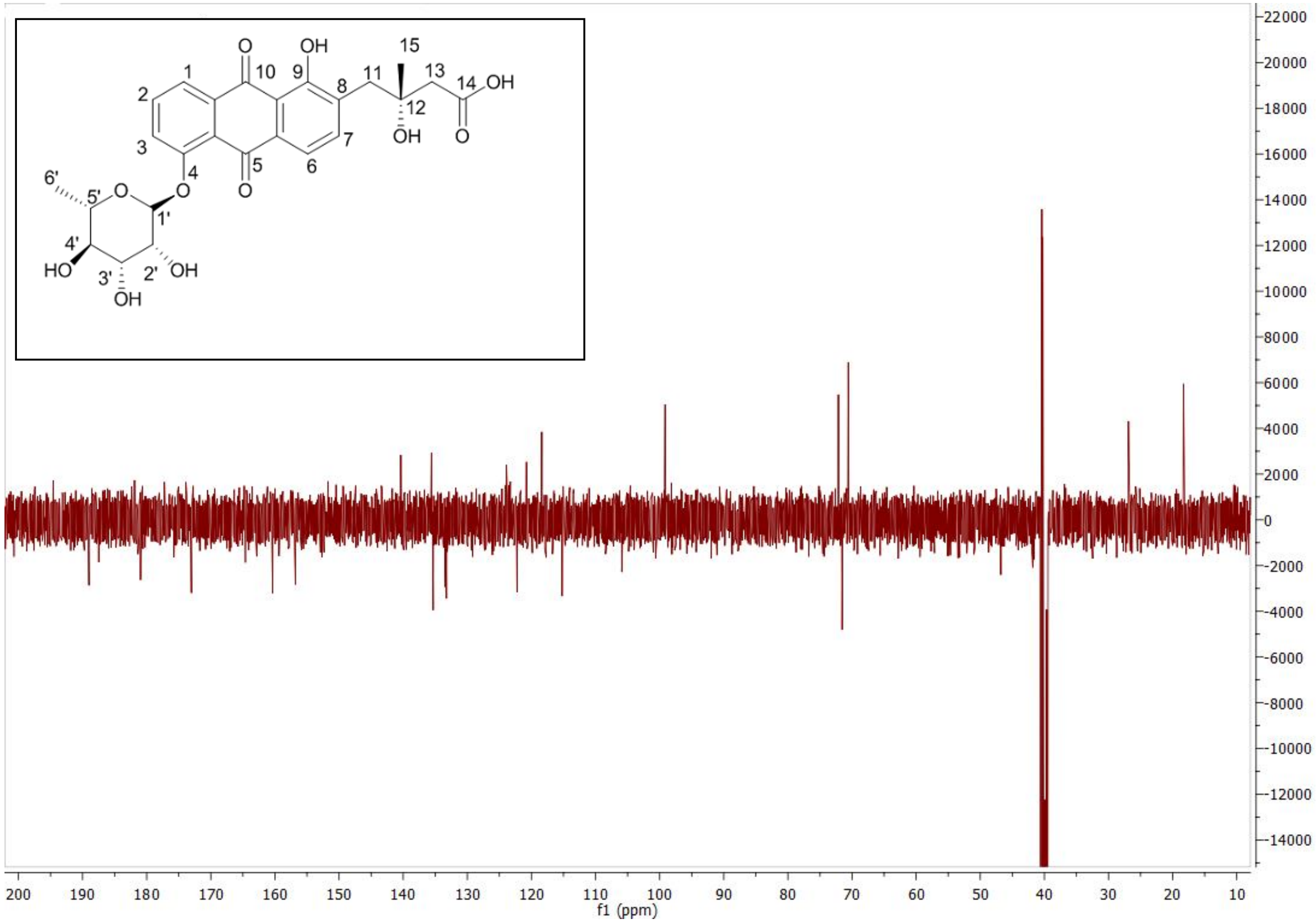

Figure S3. ^{13}C-NMR spectrum of compound 1 (Fridamycin H) in DMSO-*d6* (150 MHz)

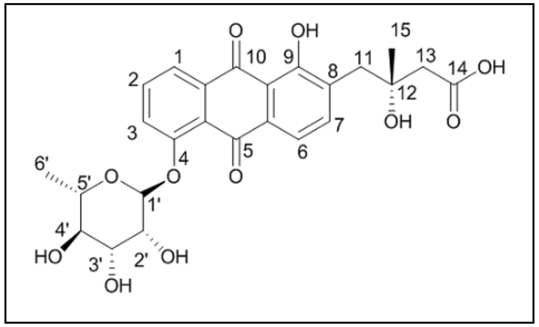

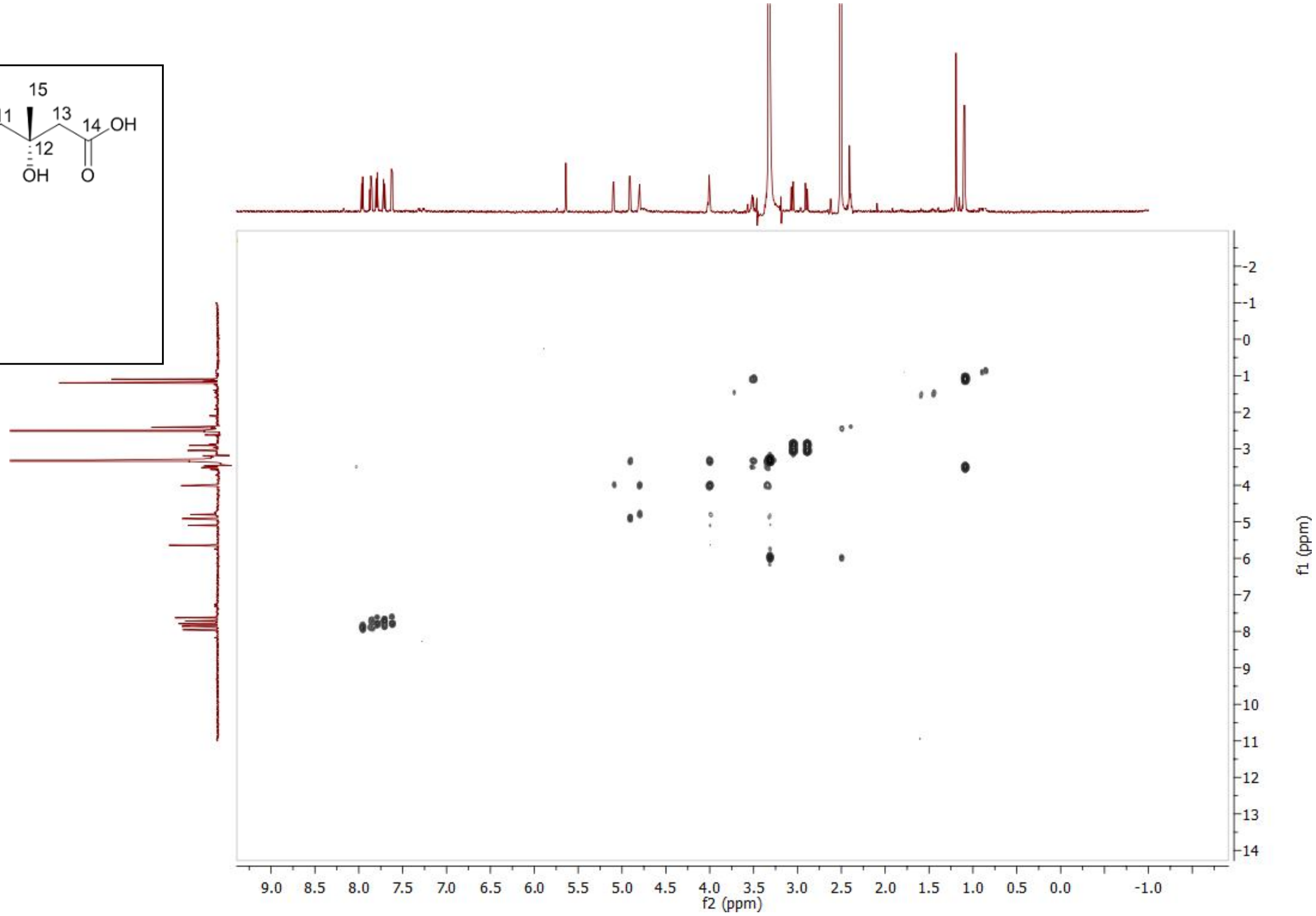

Figure S4: ^{1}H-^{1}H COSY spectrum of compound 1 (Fridamycin H) in DMSO-*d6*

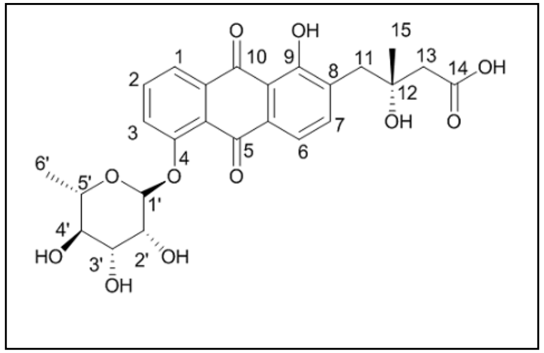

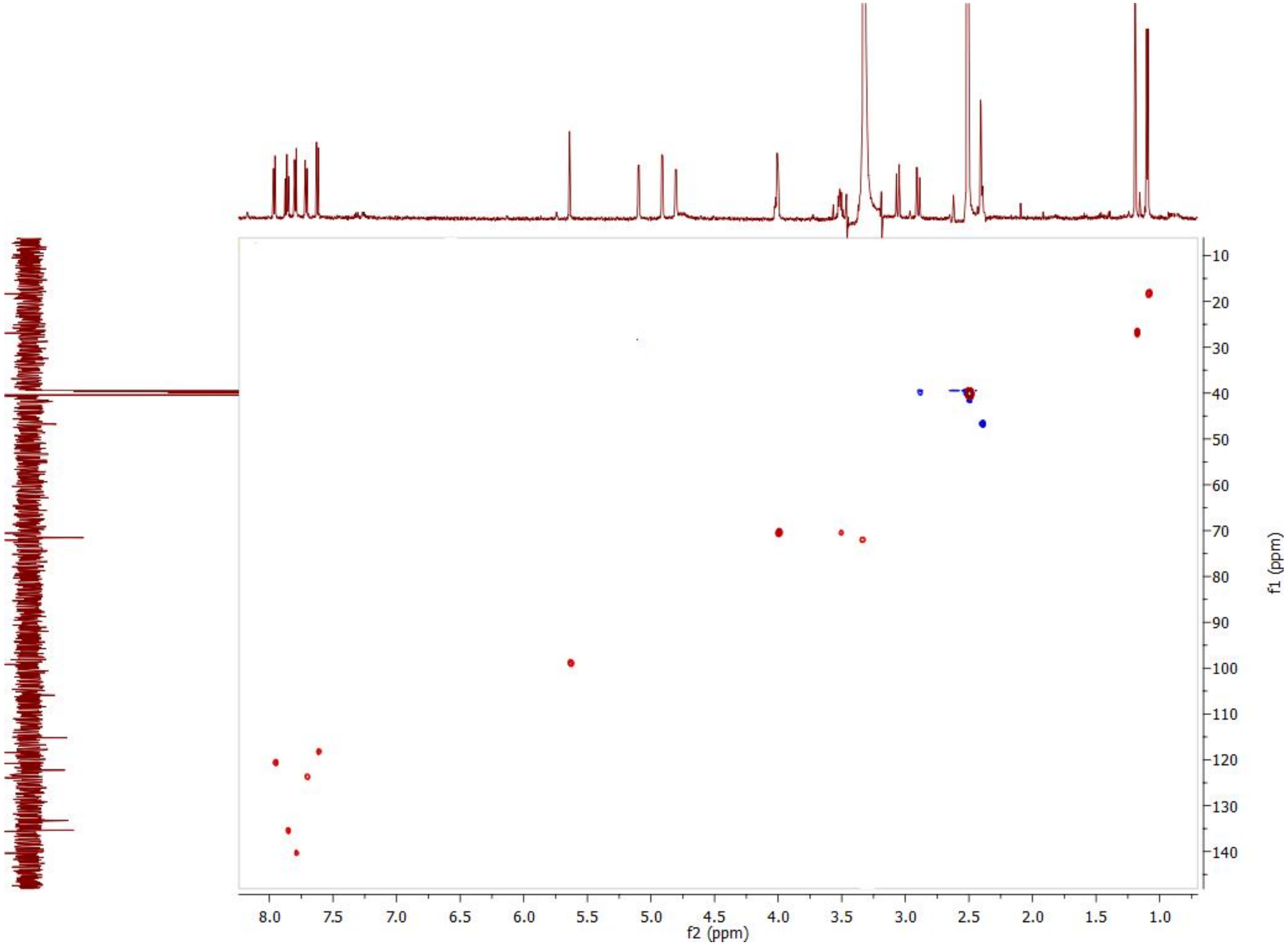

Figure S5: HSQC spectrum of compound 1 (Fridamycin H) in DMSO-*d6*

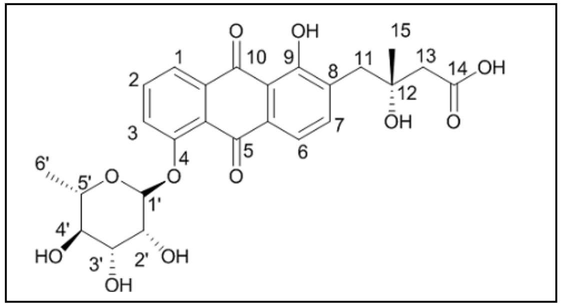

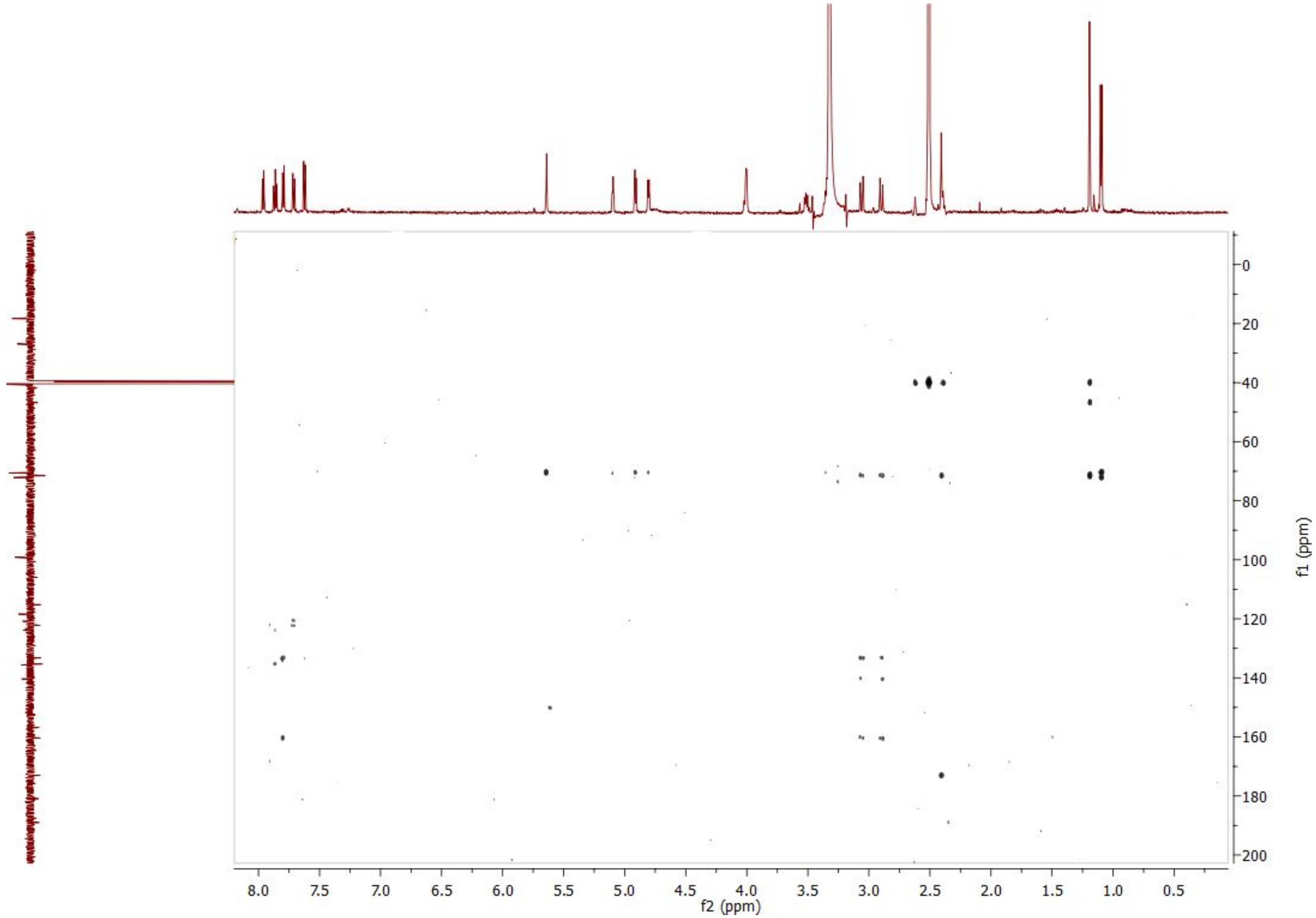

Figure S6: HMBCspectrum of compound 1 (Fridamycin H) in DMSO-*d6*

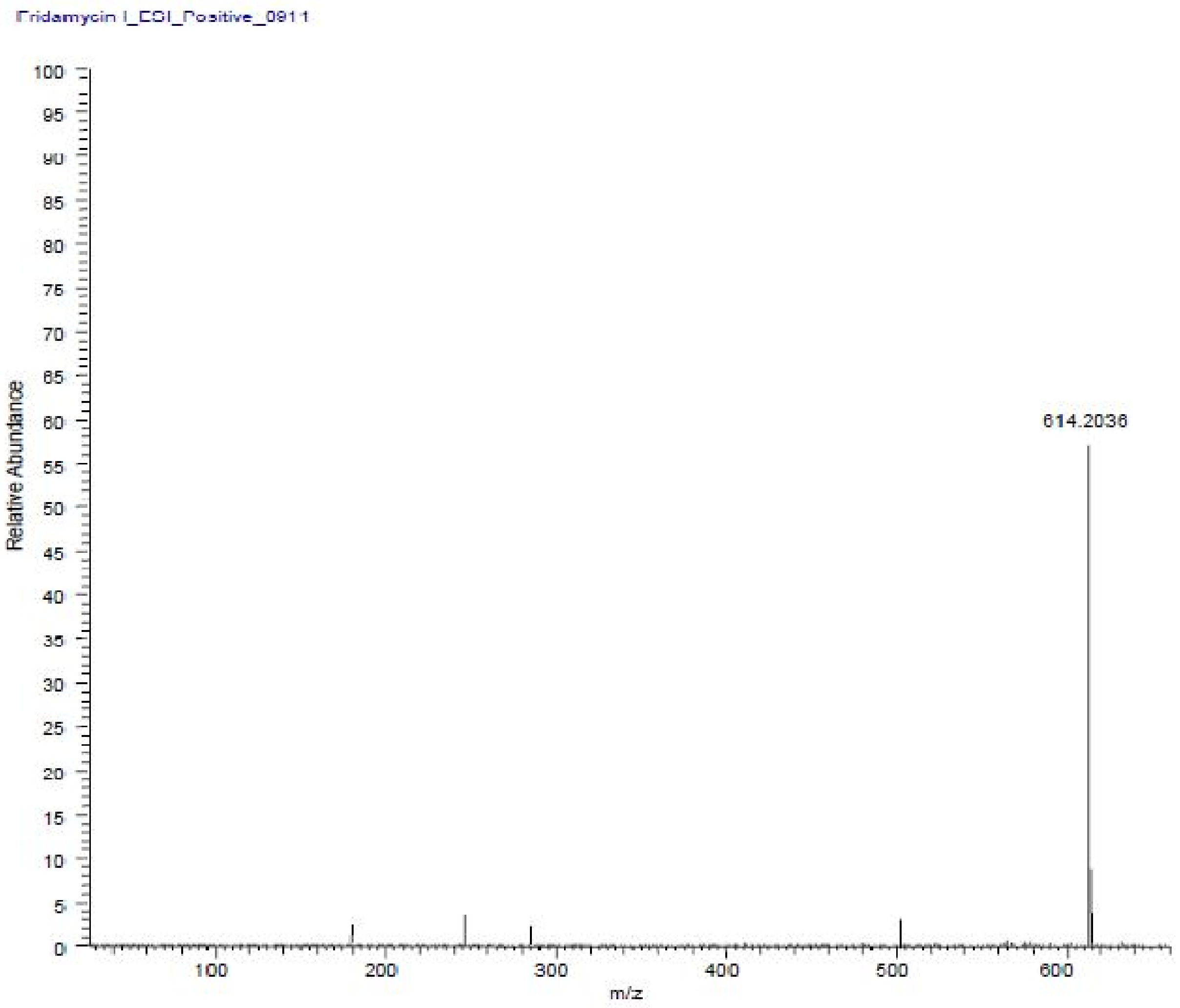

Figure S7. HR-ESIMS spectrum of compound 2 (Fridamycin I)

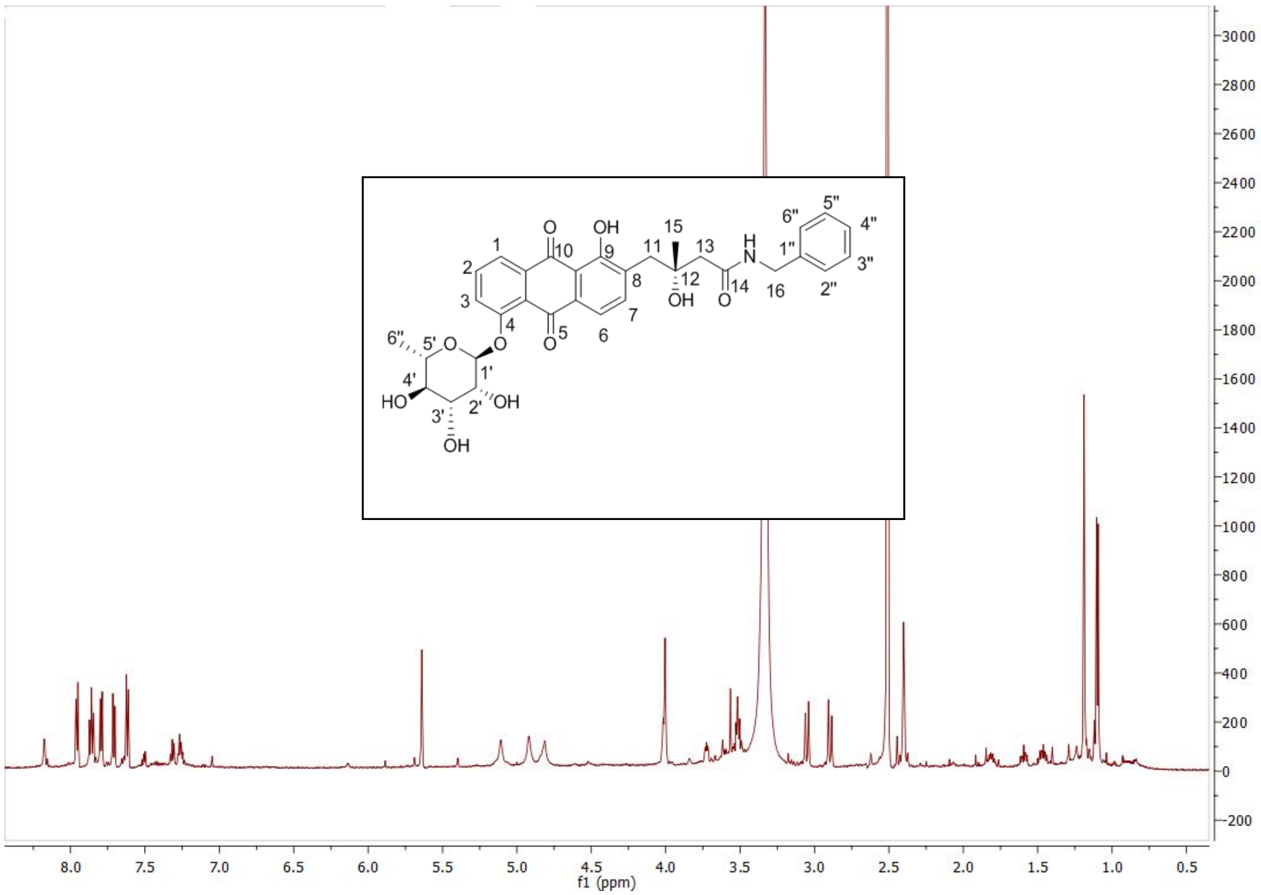

Figure S8. ^{1}H-NMR spectrum of compound 2 (Fridamycin I) in DMSO-*d*6 (600 MHz)

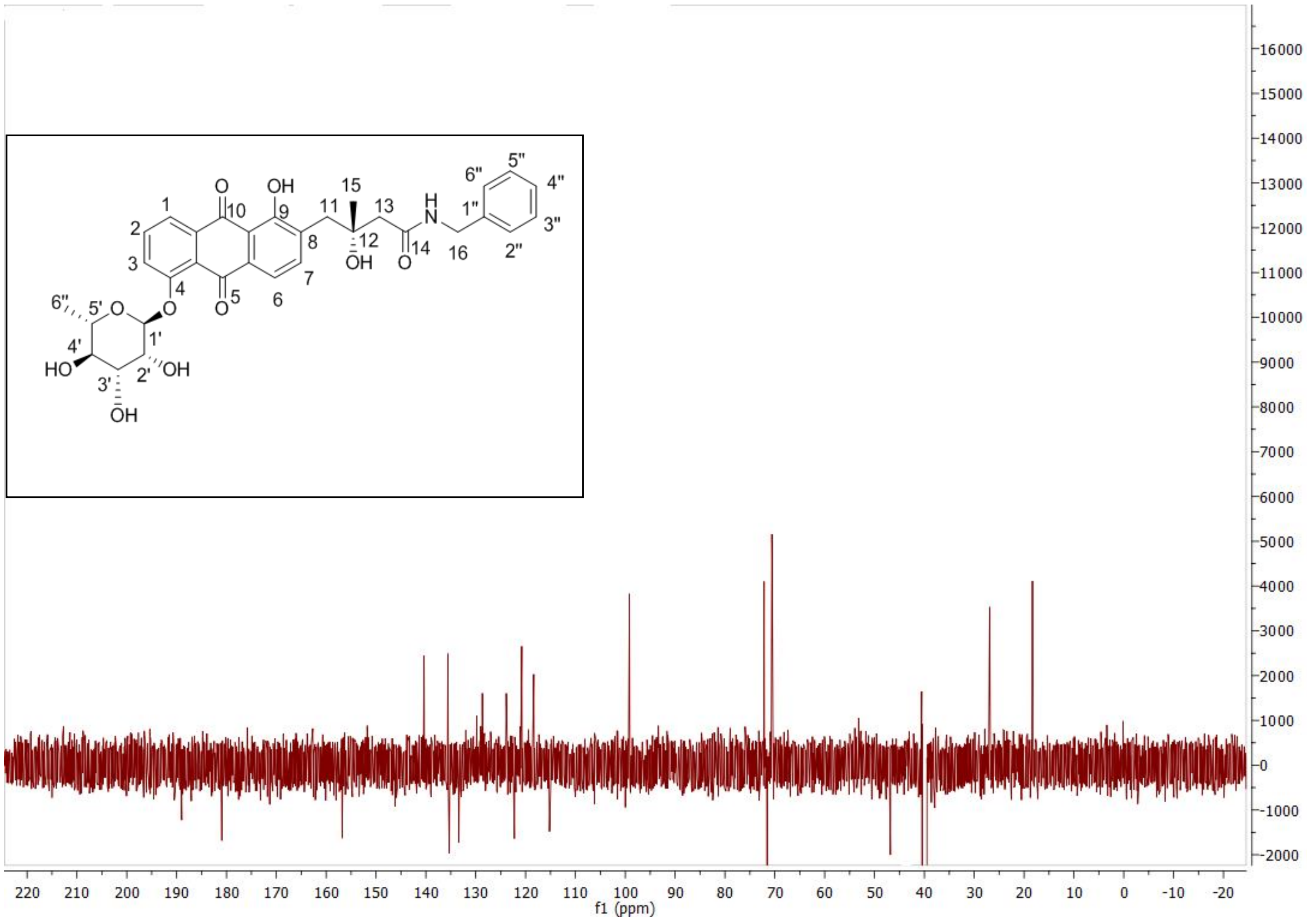

Figure S9. ^{13}C-NMR spectrum of compound 2 (Fridamycin I) in DMSO-*d6* (150 MHz)

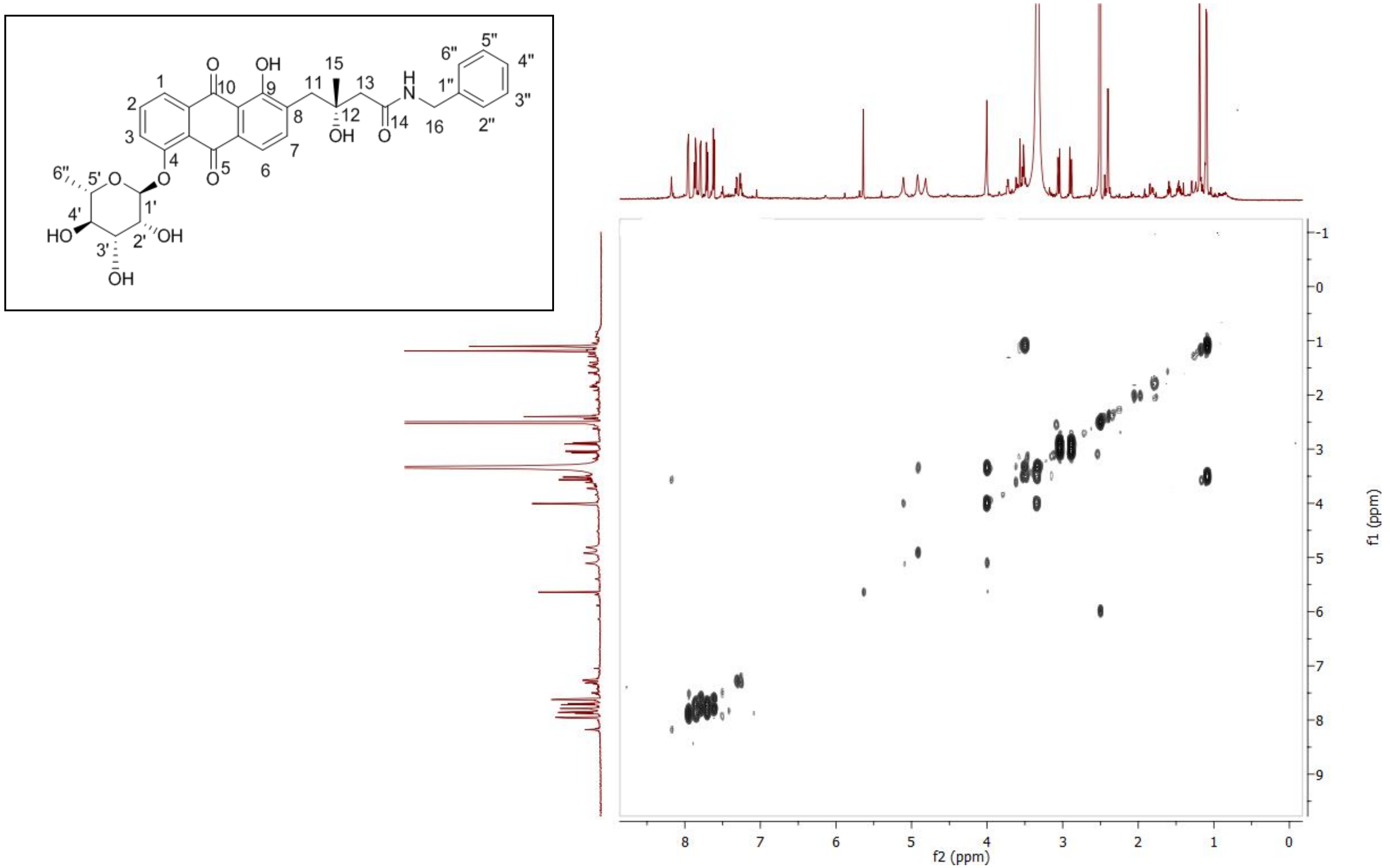

Figure S10: ^{1}H-^{1}H COSY spectrum of compound 2 (Fridamycin I) in DMSO-*d6*

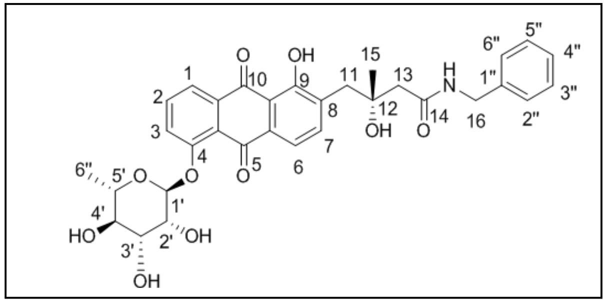

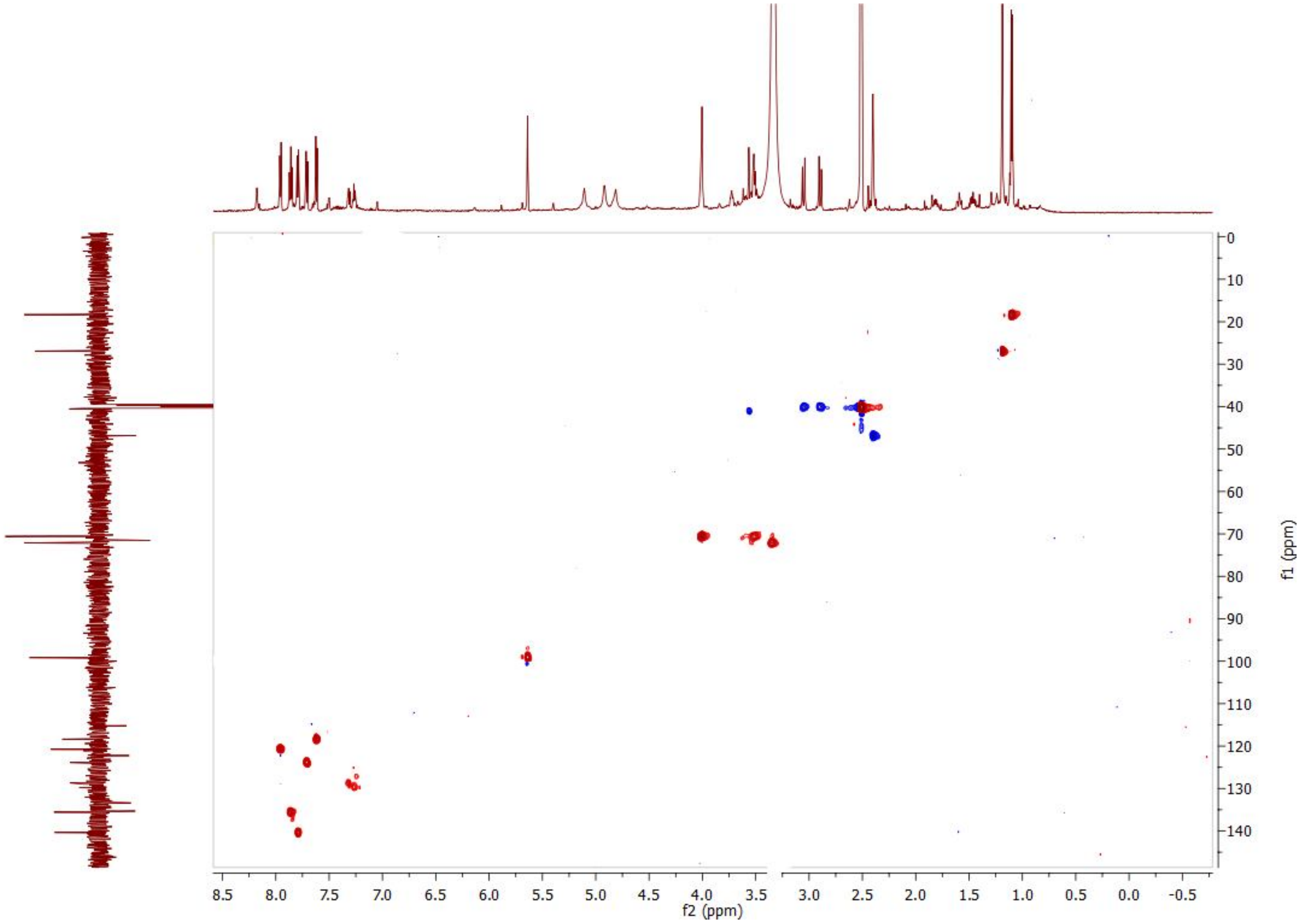

Figure S11: HSQC spectrum of compound 2 (Fridamycin I) in DMSO-*d6*

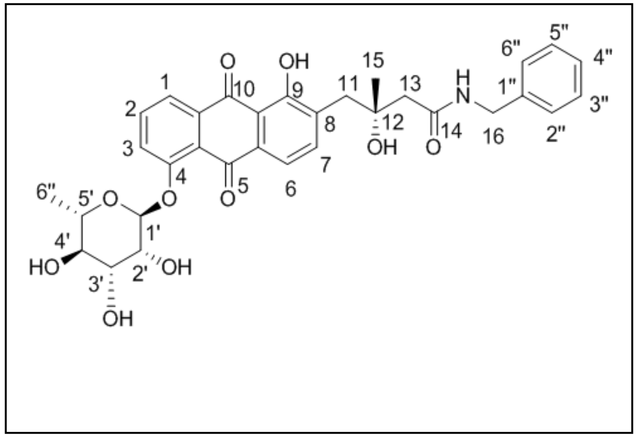

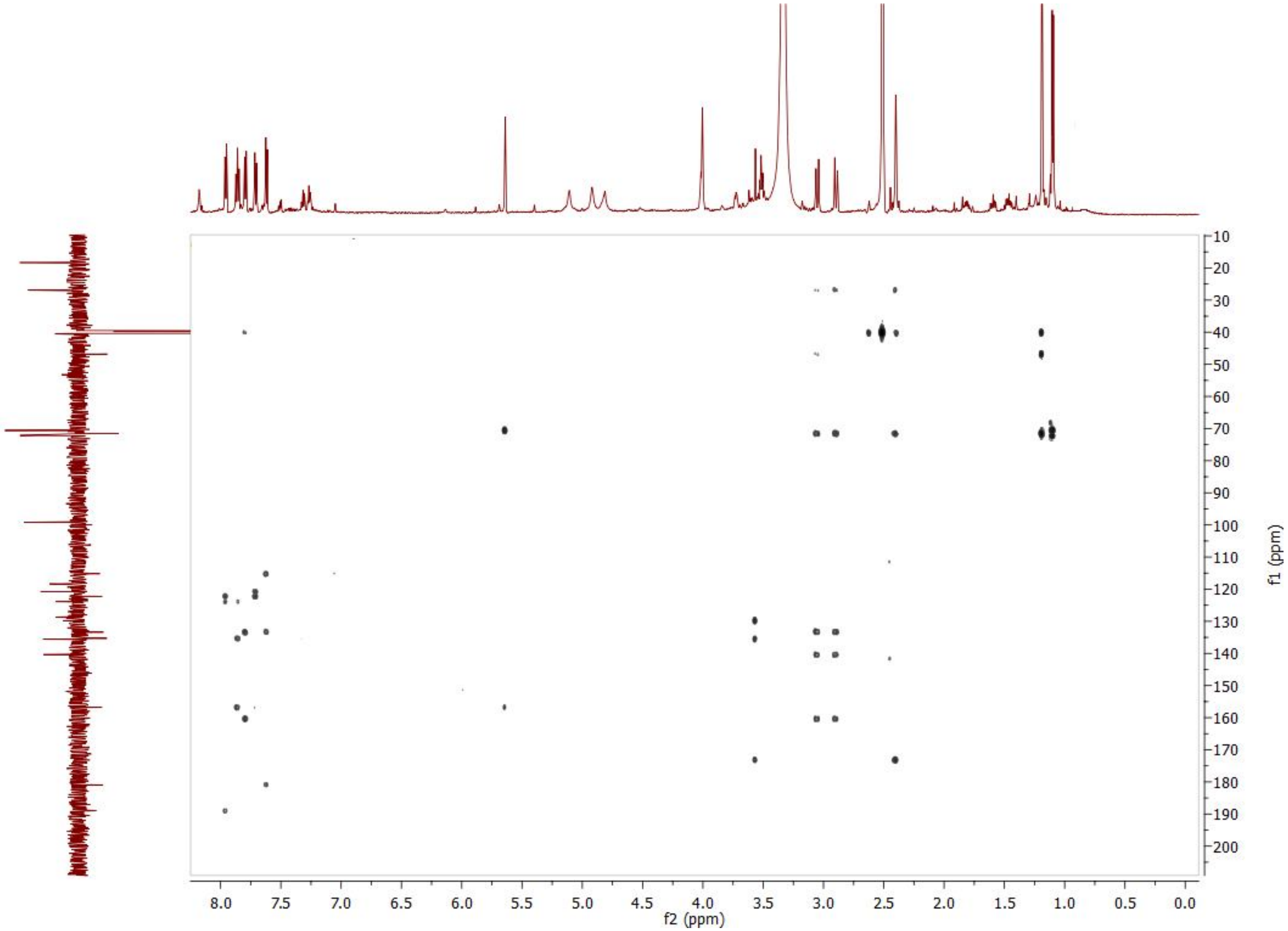

Figure S12: HMBC spectrum of compound 2 (Fridamycin I) in DMSO-*d6*

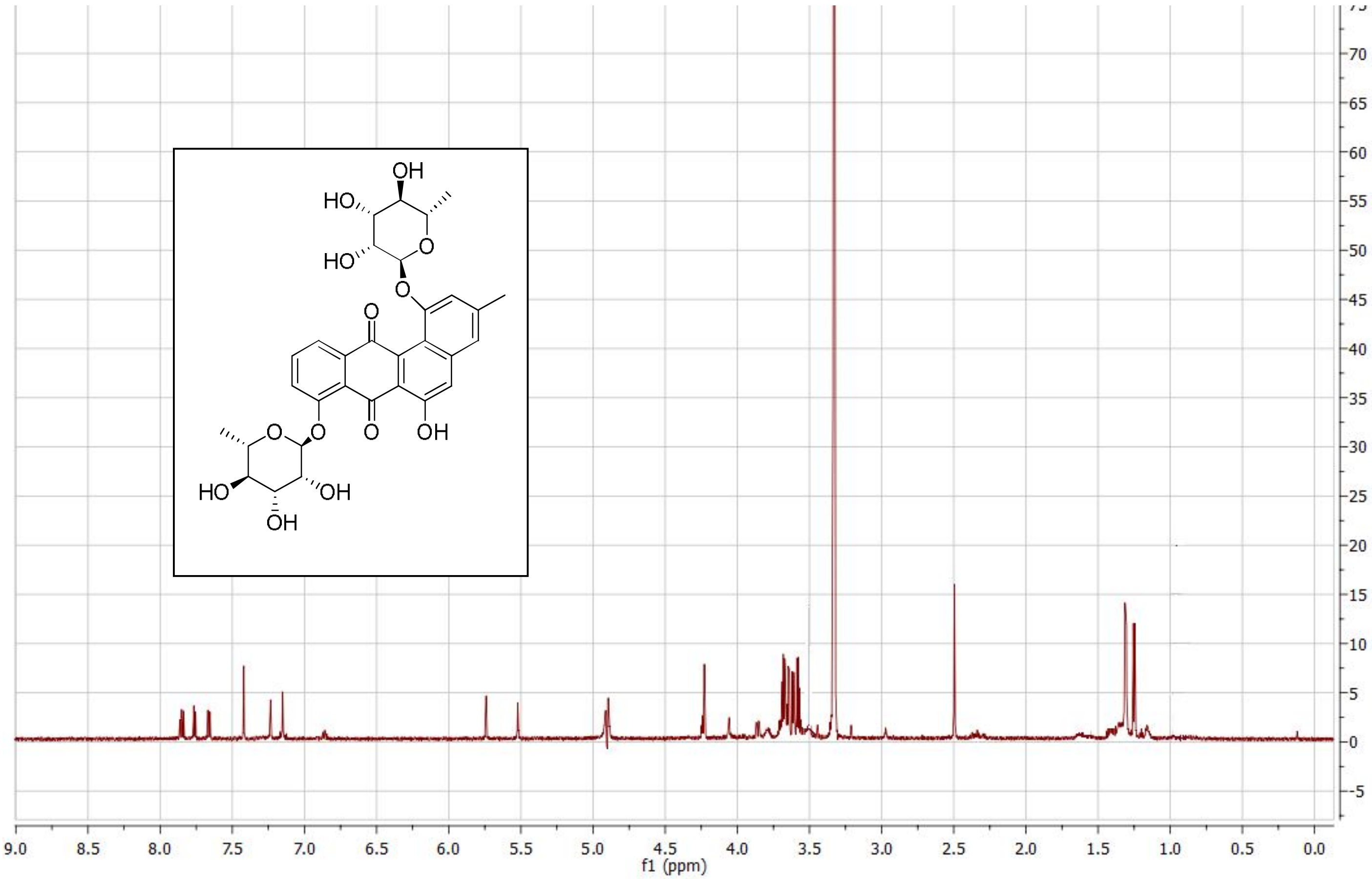

Figure S13: ^{1}H-NMR spectrum of Actinosporin C in MeOD (600 MHz)

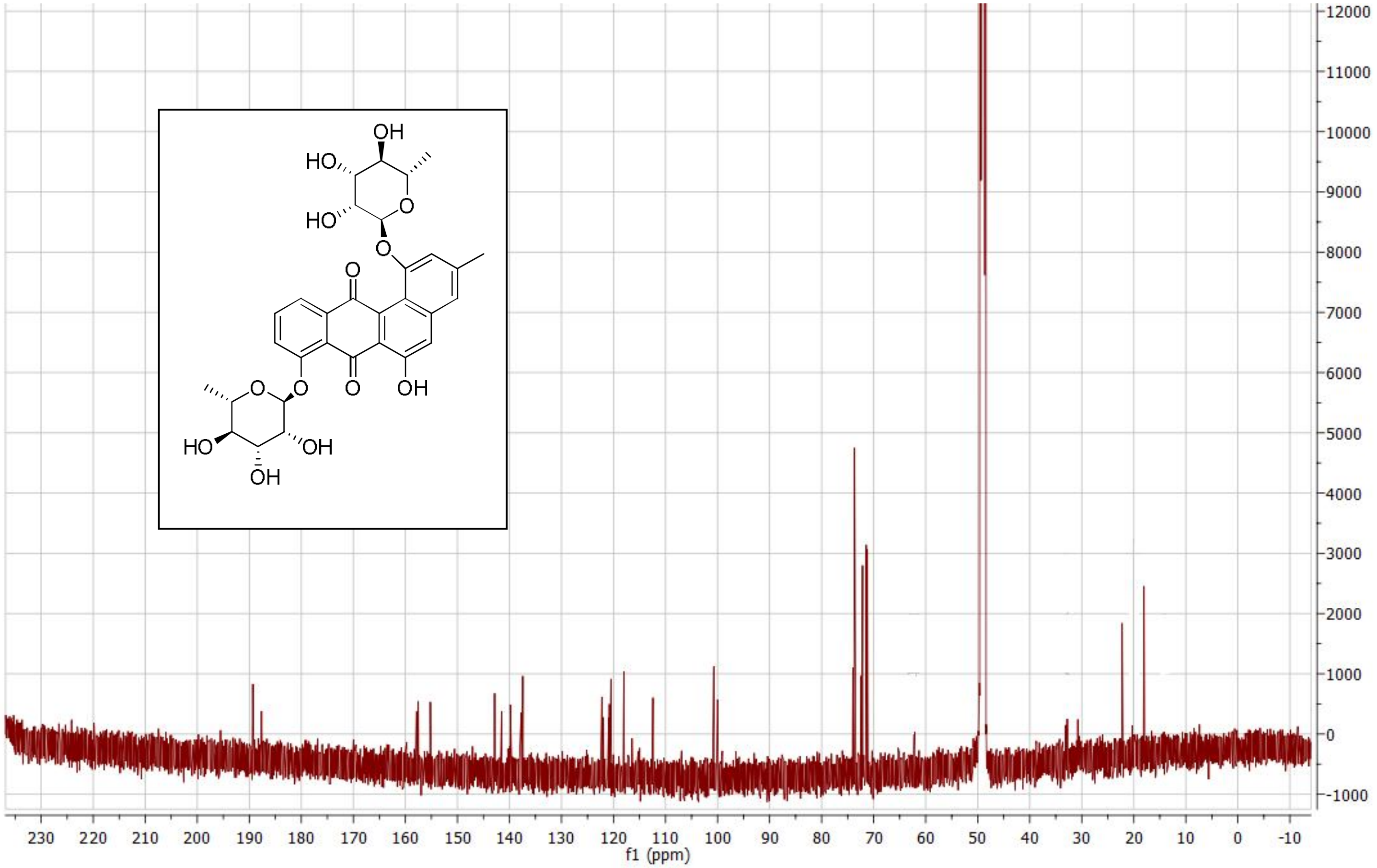

Figure S14: ^{13}C-NMR spectrum of Actinosporin C in MeOD (150 MHz)

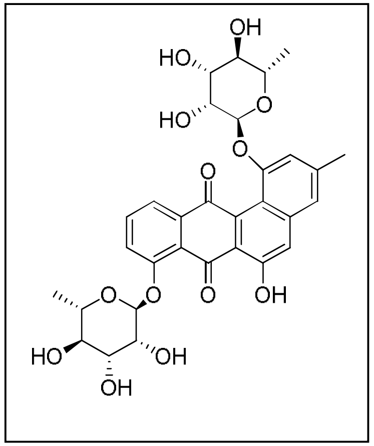

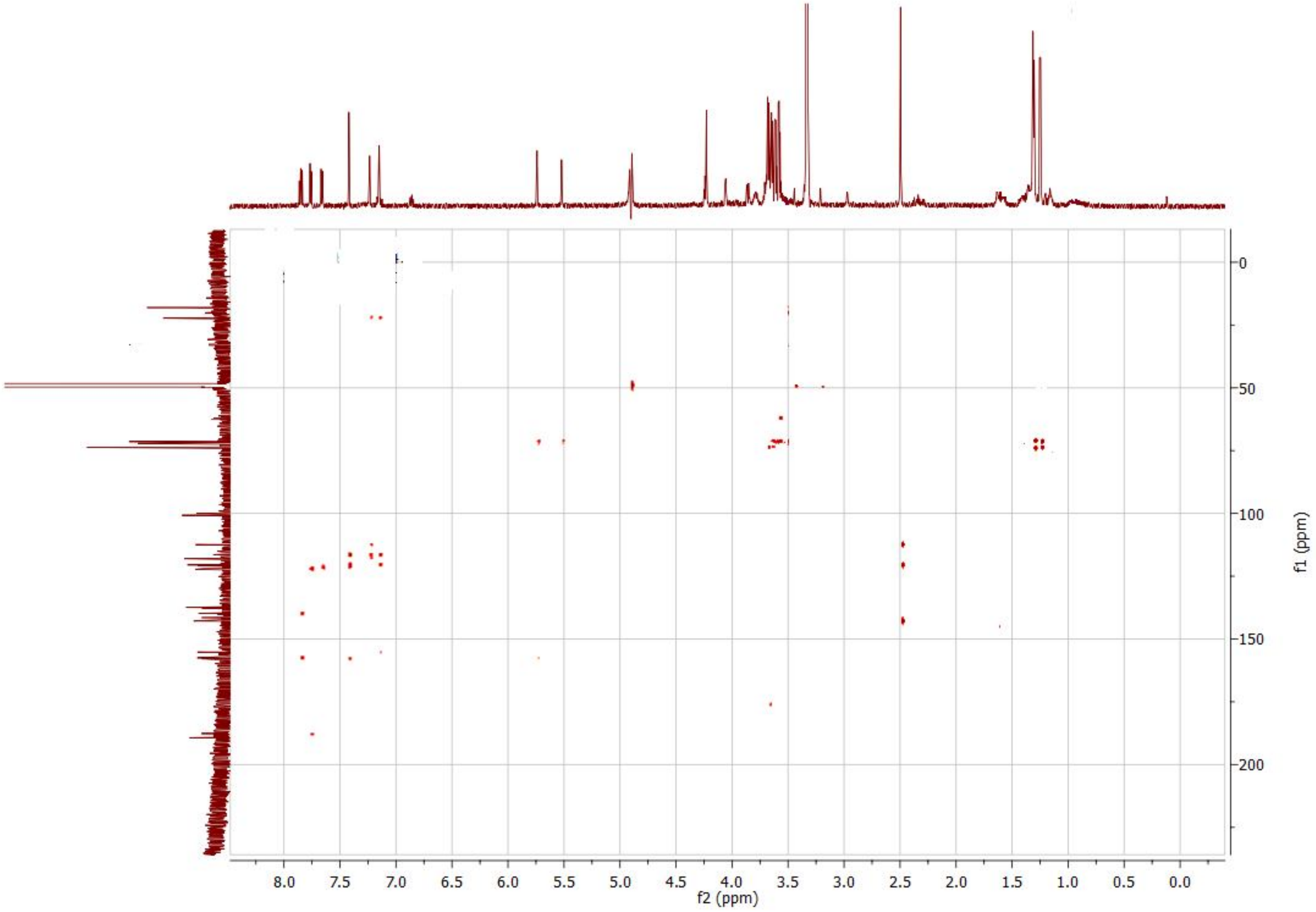

Figure S15: HBMC spectrum of Actinosporin C in MeOD

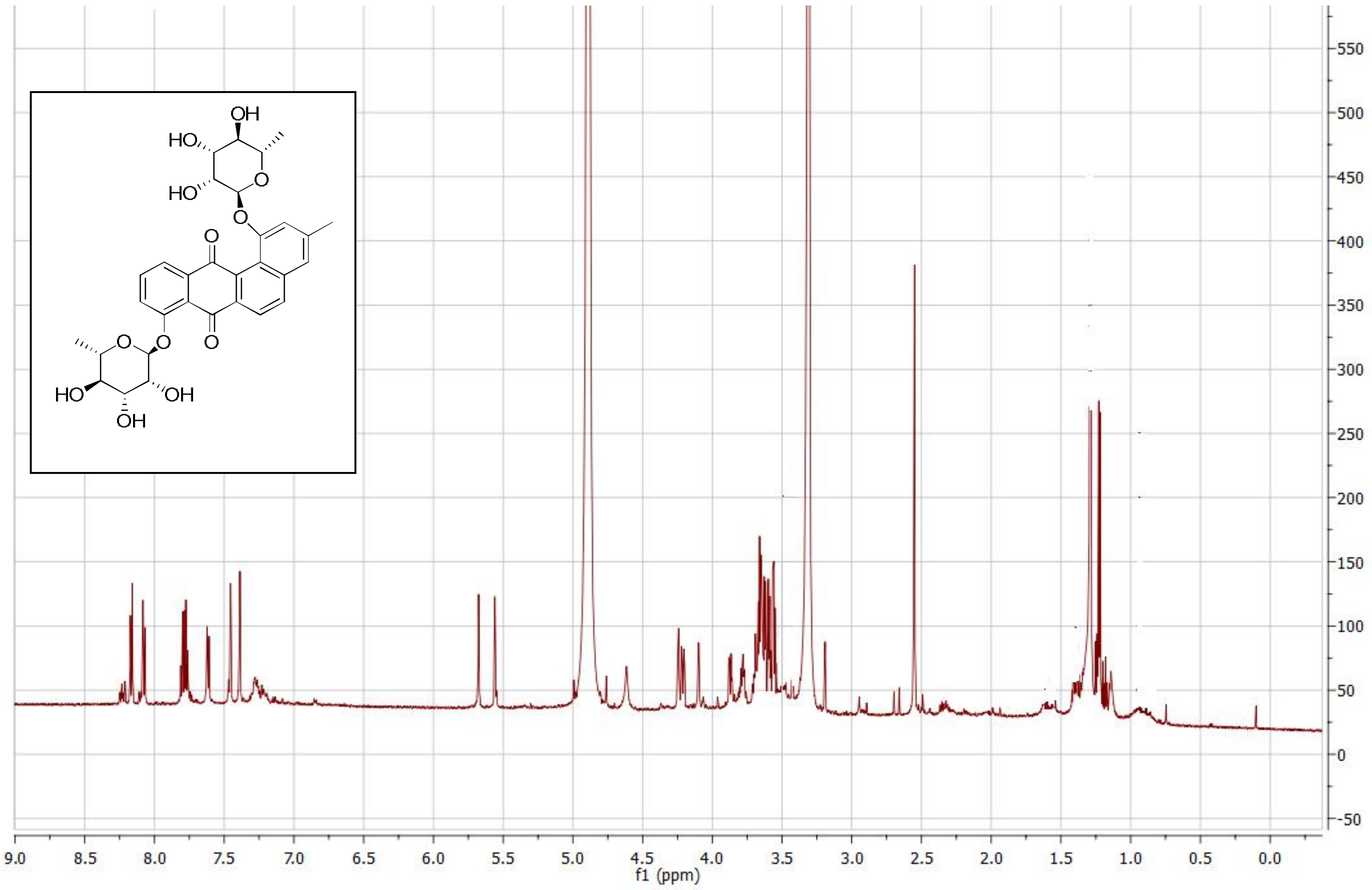

Figure S16: ^{1}H-NMR spectrum of Actinosporin D in MeOD (600 MHz)

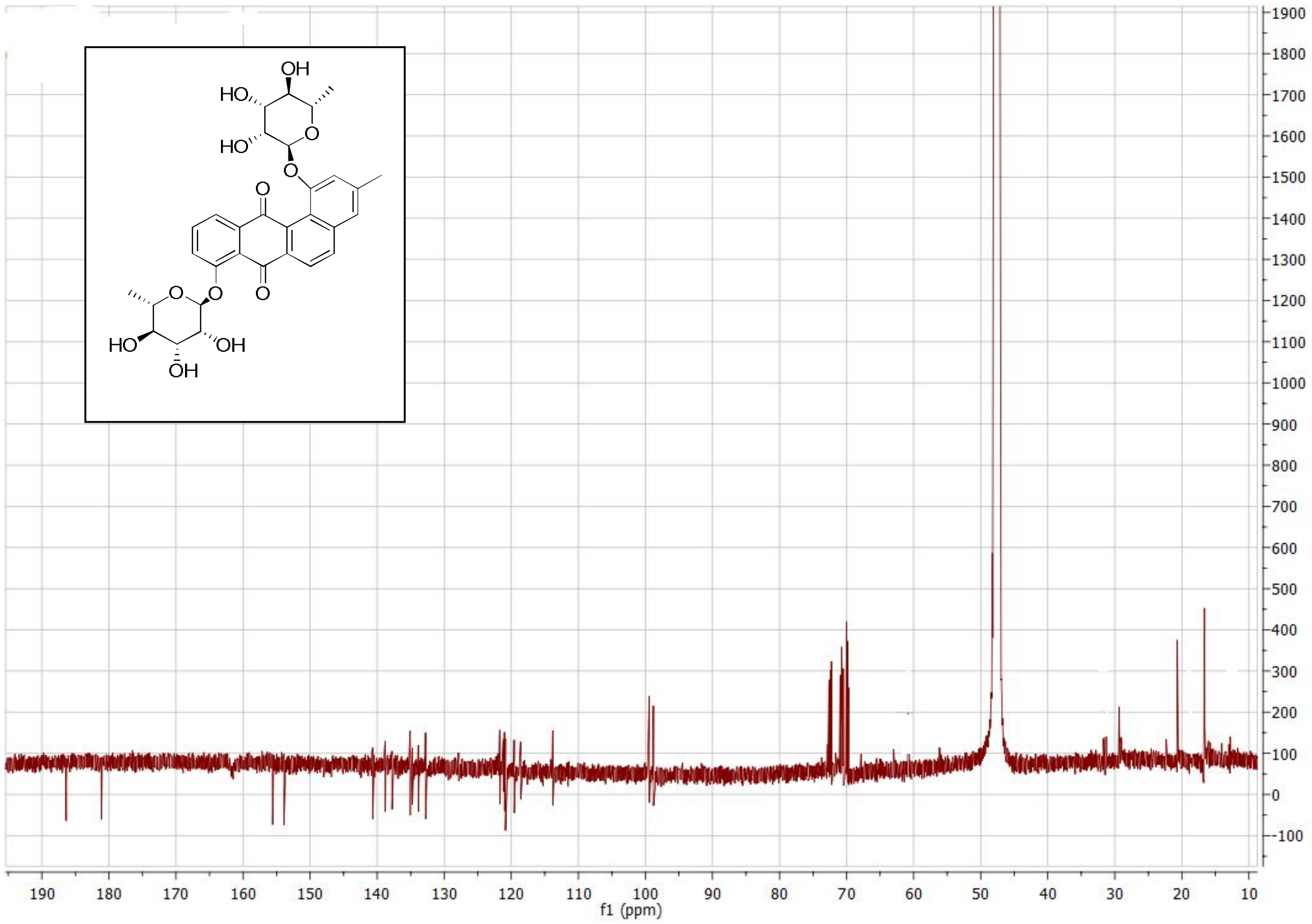

Figure S17: ^{13}C-NMR spectrum of Actinosporin D in MeOD (150 MHz)

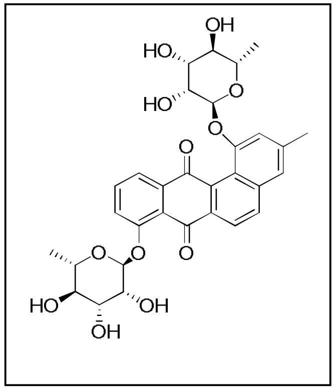

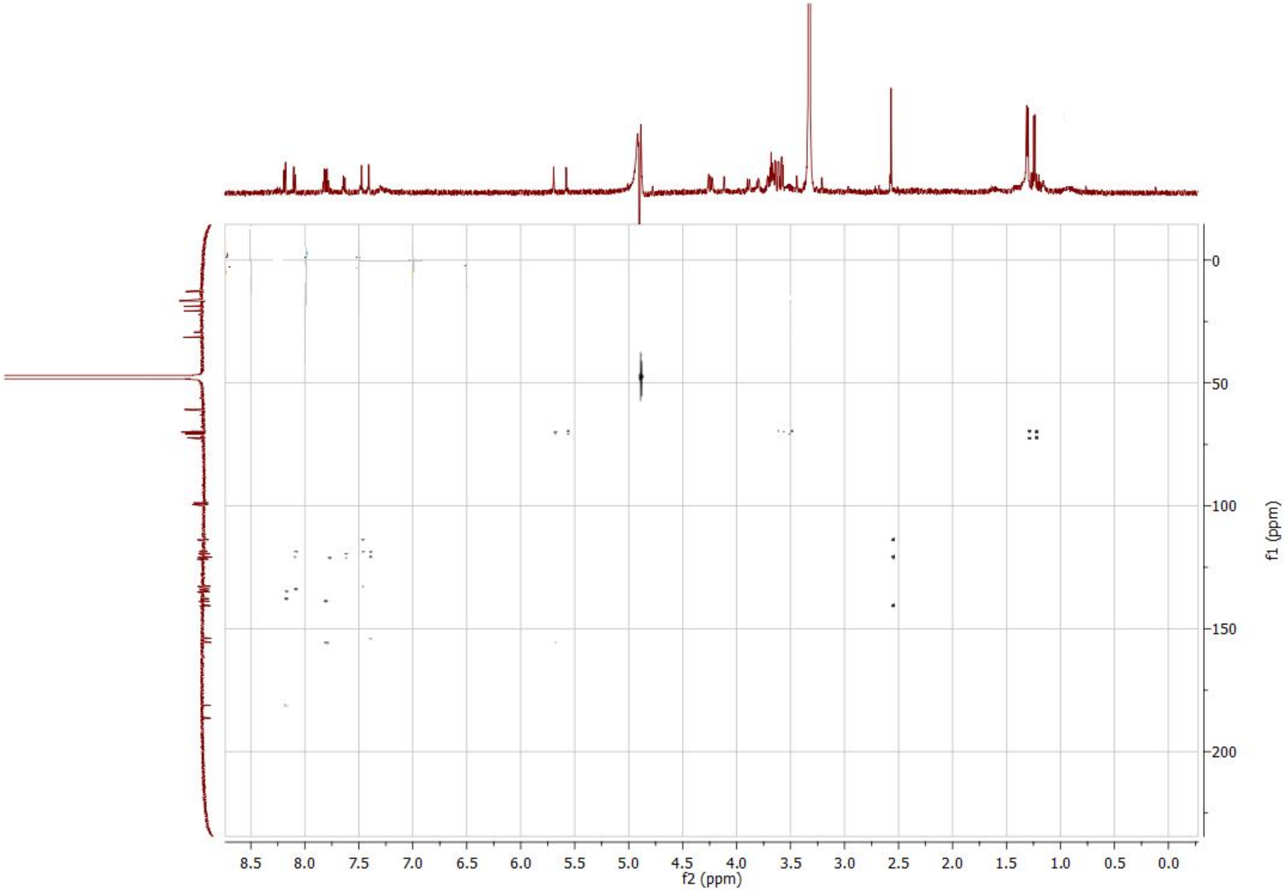

Figure S18: HBMC spectrum of Actinosporin D in MeOD

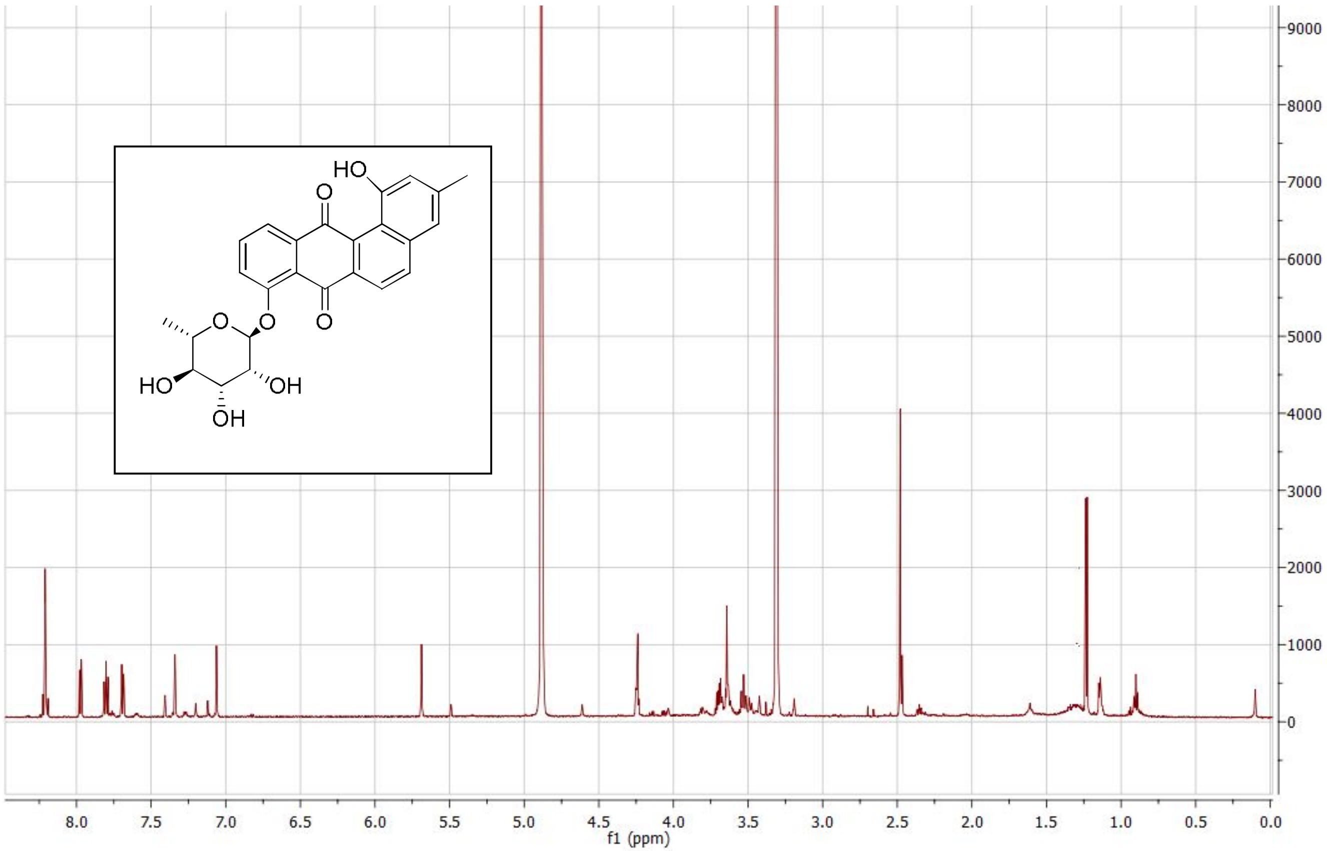

Figure S19: ^{1}H-NMR spectrum of Actinosporin G in MeOD (600 MHz)

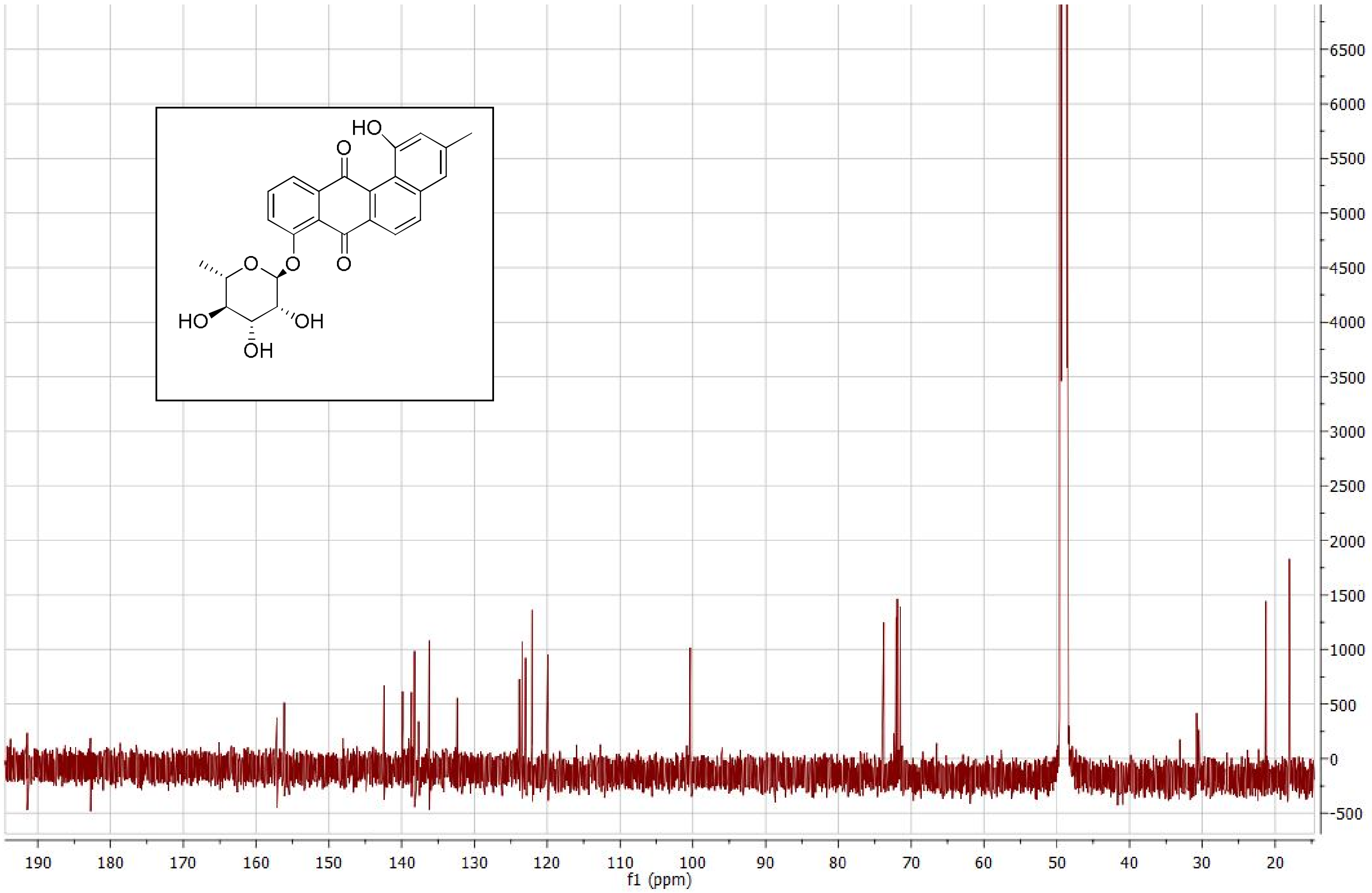

Figure S20: ^{13}C-NMR spectrum of Actinosporin G in MeOD (150 MHz)

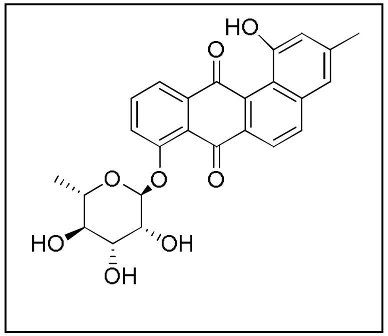

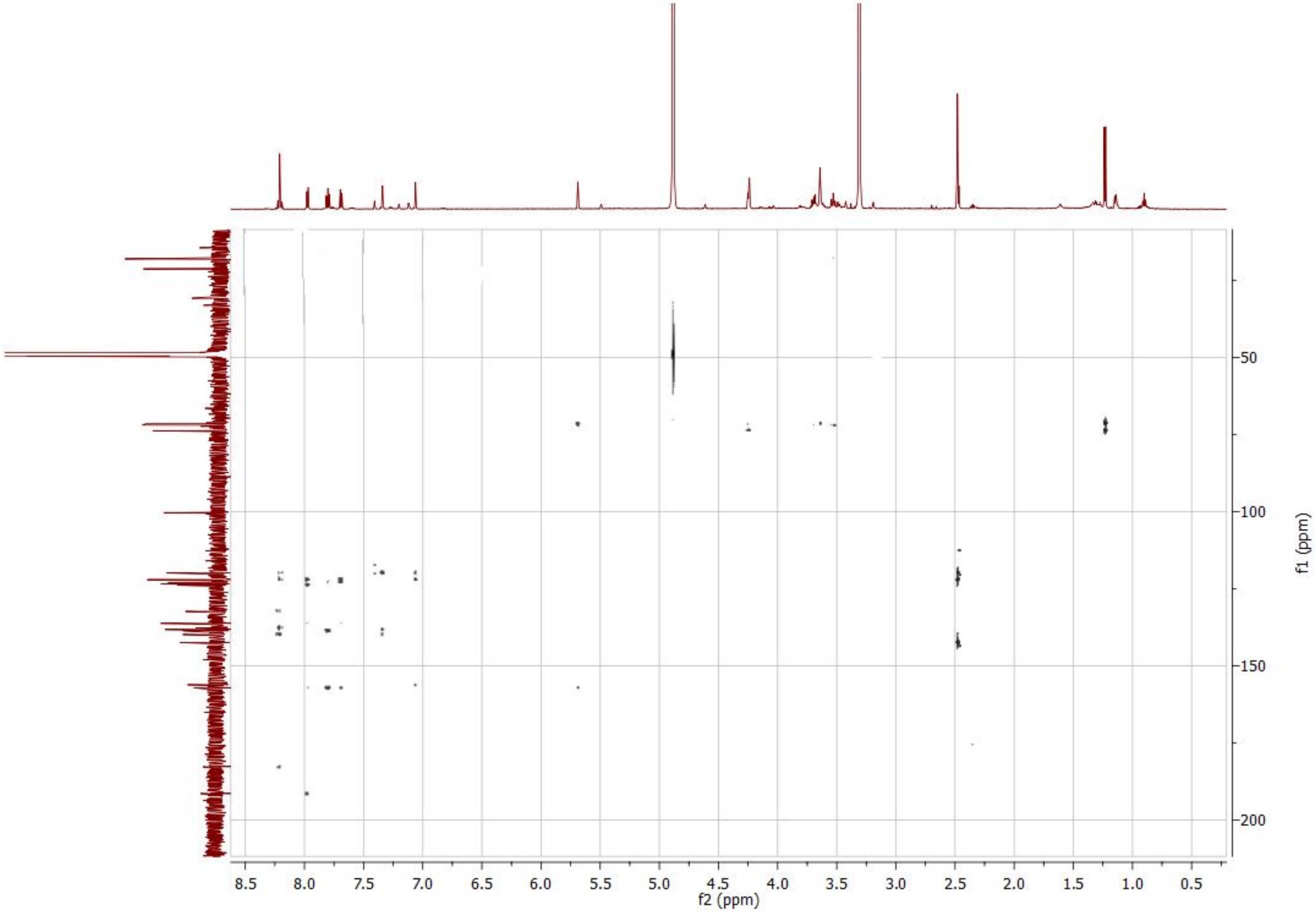

Figure S21: HBMC spectrum of Actinosporin G in MeOD
